# Recombinant humanized type III collagen improves ovarian function via ITGA2-mediated mitochondrial function restoration in granulosa cells and extracellular matrix remodeling

**DOI:** 10.1093/rb/rbag046

**Published:** 2026-03-28

**Authors:** Quan Wei, Shuaibin Liu, Zhiyong Dong, Hui Zeng, Shuang You, Jingcong Dai, Li Wang, Xia Shi, Jiashuo Liu, Jie Xing, Jiaming Chen, Xinyi Wang, Fan He, Yun Zhu, Xia Yang, Lina Hu

**Affiliations:** Department of Obstetrics and Gynecology, The Second Affiliated Hospital of Chongqing Medical University, Chongqing 400010, China; Department of Obstetrics and Gynecology, The Second Affiliated Hospital of Chongqing Medical University, Chongqing 400010, China; Department of Obstetrics and Gynecology, The Second Affiliated Hospital of Chongqing Medical University, Chongqing 400010, China; Department of Obstetrics and Gynecology, The Second Affiliated Hospital of Chongqing Medical University, Chongqing 400010, China; Department of Obstetrics and Gynecology, The Second Affiliated Hospital of Chongqing Medical University, Chongqing 400010, China; Department of Obstetrics and Gynecology, The Second Affiliated Hospital of Chongqing Medical University, Chongqing 400010, China; Chengdu Women and Children’s Central Hospital, University of Electronic Science and Technology of China, Chengdu, Sichuan 610091, China; Department of Obstetrics and Gynecology, The Second Affiliated Hospital of Chongqing Medical University, Chongqing 400010, China; Department of Obstetrics and Gynecology, The Second Affiliated Hospital of Chongqing Medical University, Chongqing 400010, China; Department of Obstetrics and Gynecology, The Second Affiliated Hospital of Chongqing Medical University, Chongqing 400010, China; Department of Obstetrics and Gynecology, The Second Affiliated Hospital of Chongqing Medical University, Chongqing 400010, China; Department of Obstetrics and Gynecology, The Second Affiliated Hospital of Chongqing Medical University, Chongqing 400010, China; The Center for Reproductive Medicine, Department of Obstetrics and Gynecology, The Second Affiliated Hospital of Chongqing Medical University, Chongqing 400010, China; Joint International Research Lab for Reproduction and Development, Ministry of Education, Chongqing 400010, China; Reproduction and Stem Cell Therapy Research Center of Chongqing, Chongqing 400010, China; National Laboratory of Biomacromolecules, Institute of Biophysics, Chinese Academy of Sciences, Beijing 100101, China; Shanxi Key Laboratory of Functional Proteins, Shanxi Jinbo Bio-Pharmaceutical Co., Ltd, Taiyuan, Shanxi 030032, China; Department of Obstetrics and Gynecology, The Second Affiliated Hospital of Chongqing Medical University, Chongqing 400010, China; Joint International Research Lab for Reproduction and Development, Ministry of Education, Chongqing 400010, China; Reproduction and Stem Cell Therapy Research Center of Chongqing, Chongqing 400010, China

**Keywords:** ovarian aging, extracellular matrix, recombinant humanized collagen, type III collagen, granulosa cell

## Abstract

Ovarian aging is characterized by depletion of the follicular reserve. The extracellular matrix (ECM) provides the essential microenvironmental niche for follicles, and aged-related alterations in ECM composition may adversely affect follicular dynamics. However, the precise relationship between matrix components and ovarian aging remains unclear. In this study, we first observed a marked reduction in type III collagen (Col III) as a key alteration associated with ovarian aging. Furthermore, we discovered that as a biomaterial, recombinant humanized type III collagen (rhCol III) ameliorated ovarian function in 12-month-old natural aging rats, evidenced by restored estrous cycles, increased ovarian index, elevated levels of anti-Müllerian hormone (AMH) and estradiol (E2) and enhanced folliculogenesis. Notably, rhCol III promoted the reorganization of the disorganized ovarian ECM into a highly ordered and dense structure, providing a supportive biological scaffold for follicular development. Additionally, supplementation of rhCol III could mitigate oxidative stress and mitochondrial dysfunction in aged granulosa cells (GCs), thereby preventing apoptosis and cellular senescence. Mechanistically, rhCol III bound to the integrin α2 (ITGA2) receptor on GCs and activated the PI3K/Akt signaling pathway. Our findings highlight Col III deficiency as a critical factor in ovarian aging and demonstrate that rhCol III supplementation rejuvenates the ovarian microenvironment via the ITGA2-PI3K/Akt axis, presenting a novel therapeutic strategy. Thus ITGA2 may represent a promising target for mitigating ovarian aging.

## Graphical Abstract

**Graphical Abstract:**
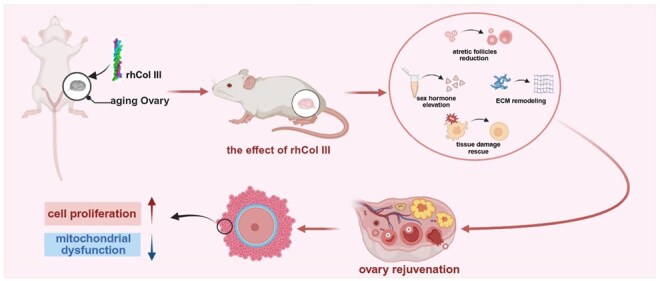

## Introduction

The ovary is a critical endocrine organ that orchestrates both the production of fertilizable oocytes and the release of key steroid hormones. Several studies have indicated that female ovarian function declines at the aged of 30 years, accelerates after the aged of 35 years, and reaches cessation at approximately the aged of 50 years [1, 2]. Meanwhile, a global trend toward delayed childbearing has increased the number of women who experienced their first pregnancy after the aged of 30 years [3]. Consequently, aged-related infertility has emerged as a challenge to female fertility. Ovarian dysfunction can also result in endocrine imbalance, contributing to the onset of menopause and increasing the risk of cardiovascular diseases [4]. Thus, mitigating ovarian aging is critically important for extending reproductive longevity and reducing the burden of aged-related diseases in aging societies.

The functional unit of the ovary is the follicle, which is surrounded by the ovarian stromal microenvironment consisting of a variety of cell types and extracellular matrix (ECM) [5]. In the ovary, the ECM provides a structural scaffold and biochemical signals for follicles. The stability of ECM complex composition and structure is crucial for follicular survival and ovarian function [6]. Alterations of ECM components contribute to the biomechanical changes in the perifollicular microenvironment, affecting follicular quiescence or activation [7].

Collagen is a major component of the ECM and the most abundant ECM protein in mammals [8]. Its various inherent properties, such as mechanical properties and cell pathway signals, contribute to the maintenance of a matrix microenvironment for cells [9]. In the collagen family, 29 genetically distinct types of collagen have been described so far [10]. Collagen is expressed in many organs, including the skin, joints and internal organs. Among all types of collagen, type I collagen (Col I) and type III collagen (Col III) represent the largest share, making up to 80–90% of the total collagen in the human body [10]. Col I, Col III and type IV collagen (Col IV) are distributed in the ovaries. Among them, Col III is particularly found in the matrix around the follicle [11]. Abnormal expression of collagen is closely associated with follicular dysplasia. Ouni et al. reported that compared to adolescent females, collagen expression significantly increased in the ovaries of women of reproductive aged [12]. Saha et al. demonstrated that hypothyroidism led to ovarian dysfunction and reproductive disorder mainly by reducing collagen synthesis in female rats [13].

The close structural and functional association between collagen and follicles makes collagen a promising candidate material for follicle encapsulation strategies. Joo et al. demonstrated that a collagen-rich 3D system could promote oocyte maturation and maintain the production of steroid hormones [14]. Yang et al. [15] transplanted collagen scaffolds into the ovaries of mice with premature ovarian failure (POF) and found increased sex hormone levels in the collagen scaffold group. However, collagen derived from animal tissues increases the risk of immune reactions and viral transmission. Recombinant humanized type III collagen (rhCol III) minimizes the risk of immunogenicity as its sequence is entirely derived from specific human type III collagen gene fragments [16]. rhCol III produced by biosynthesis technology exhibits higher water solubility and bioactivity. Our previous studies have demonstrated that rhCol III possessed favorable biocompatibility and strong cell-adhesion capacity [17]. It promoted the regeneration of atrophied vaginal epithelium [18], restored the immune microenvironment of the endometrium and improved the outcomes of pregnancy [19, 20].

Given the critical role of Col III in follicular development and function, supplementation with rhCol III represents a promising therapeutic strategy for rescuing ovarian function. Therefore, we explored the relationship between the deficiency of Col III and ovarian function decline. To assess the effects of rhCol III on ovarian aging, a naturally aging rat model was employed to investigate the efficiency and safety of rhCol III for restoring ovarian function. Furthermore, an aging granulosa cell (GC) model was established to explore how rhCol III restores ovarian reserve.

## Materials and methods

### Preparation of rhCol III

The rhCol III employed in this study was constructed based on the Gly483-Pro512 fragment derived from the human *COL3A1* chain, which comprises 16 tandem repeats of a triple-helical motif. The amino acid sequence of rhCol III was previously validated and was publicly available in the Protein Data Bank (PDB) database under the numbers 6A0C and 6A0A. rhCol III was produced following a previously published study. The lyophilized protein was reconstituted in sterile phosphate-buffered saline (PBS) before experimentation. For morphological characterization, the samples were observed by scanning electron microscopy (SEM).

### Human sample collection

In total, 20 human ovarian tissue samples were collected, composed of two groups: the young group (*n* = 10; aged range: 25–38 years) and the aged group (*n* = 10; aged range: 40–55 years). All tissues were obtained from patients undergoing laparoscopic surgery for benign ovarian conditions. Following collection, the tissue specimens were fixed, dehydrated and embedded in paraffin for further experiments. Ethics approval for the study was obtained from the Ethics Committees of the Second Affiliated Hospital of Chongqing Medical University (No. 2023-91). Written informed consent was obtained from all participants.

### Cell experiments

#### Human follicular fluid collection

Women who underwent IVF-ET due to male or tubal factor infertility were included. The exclusion criteria were as follows: polycystic ovary syndrome, premature ovarian insufficiency, a history of ovarian malignancies and other endocrine disorders affecting follicular development. All included patients underwent ovarian stimulation via an antagonist protocol. Oocytes were retrieved through the vagina under ultrasound guidance 36 h after administering human chorionic gonadotropin. After oocyte isolation from the aspirate, the remaining follicular fluid was used for GC extraction.

#### Isolation and identification of human primary GCs

Human primary GCs were isolated from follicular fluid following the protocol described by a previous study [21]. Briefly, follicular fluid was centrifuged at 1500 rpm for 10 min. The pellet was resuspended in 1 mL PBS and underlaid with 2 mL of 50% Percoll solution for density gradient purification. After sequential centrifugation, purified cells were obtained after 10-min digestion with 0.25% trypsin at 37°C. Isolated GCs were cultured in the DMEM/F12 medium containing 10% FBS. Cell identification was conducted using immunofluorescence staining against the cell-specific marker FSHR (Supplementary Figure S1). Collected GCs were grouped by patients’ aged: the young group (*n* = 10; aged range: 24–38 years) and the aged group (*n* = 10; aged range: 39–44 years). Protein extraction was performed when cells reached 80% confluence.

#### Cell culture

KGN cells (a human granulosa-like tumor cell line) were obtained from Procell Life Science & Technology Co., Ltd (Wuhan, China). Both KGN cells and GCs were cultured in the F12 medium containing 10% FBS and 1% penicillin-streptomycin at 37°C in a 5% CO_2_ humidified incubator.

#### Establishment and identification of aged GC model

A replicative senescence model of GCs was constructed through serial passaging. Cells at passage 50 and beyond were identified as senescent cells based on elevated expression levels of P21 and positive β-galactosidase staining, a model widely adopted in previous studies [21, 22]. In this study, aged GCs were used in all experiments.

A β-galactosidase staining assay was performed to evaluate cellular senescence following the manufacturer’s guidelines. Briefly, cells were added to 12-well plates with 2 × 10^5^ cells per well. After fixation with the provided fixative solution, cells were incubated with the staining solution at 37°C overnight. Senescent cells were identified using an optical microscope.

The protein expression level of P21 was quantified by western blotting. Full methodological details are available in the Western blotting subsection.

#### CCK-8 assay

KGN cells were subjected to the CCK-8 assay for proliferation analysis. Cells were seeded in 96-well plates at a density of 5000 cells/well and processed with rhCol III for 24 h. Subsequently, CCK-8 working solution was added to the well, followed by a 1-h incubation. Quantification was performed by measuring the optical density (OD) at 450 nm via a microplate reader.

#### EdU assay

GCs were seeded in 48-well plates (8 × 10^4^ cells/well) for EdU assay (Beyotime, C0071S). Following the manufacturer’s instructions, cells were incubated with 70 μL EdU labeling solution at 37°C for 30 min. Following fixation with 4% paraformaldehyde and permeabilization with 0.5% Triton X-100, the Click reaction cocktail was applied. Cell nuclei were counterstained with DAPI solution, and proliferating cells were visualized under a fluorescence microscope.

#### ROS detection

Intracellular ROS levels were detected using the reactive oxygen species (ROS) assay kit (Beyotime, S0033S). This kit works through the oxidation of DCFH-DA to DCF, which emits green fluorescence when excited at 488 nm. After incubation with DCFH-DA, following PBS washes, the cells were immediately examined under a Nikon fluorescence microscope. Mean fluorescence intensity was determined with background subtraction using the ImageJ software.

#### Antioxidant enzyme assays

Intracellular glutathione (GSH) levels were quantified using a commercial kit (Solarbio, BC1175) according to the manufacturer’s protocol. This assay is based on the reaction of GSH with 5,5′-dithiobis-(2-nitrobenzoic acid) (DTNB) to generate 2-nitro-5-thiobenzoic acid (TNB), which exhibits maximal absorbance at 412 nm. After incubation, the OD was recorded at 412 nm using a microplate reader.

Similarly, the activity of superoxide dismutase (SOD) was determined using a detection kit (Solarbio, BC5165). The assay employs WST-8 formazan formation kinetics, where SOD-mediated inhibition of superoxide anions reduces chromogen production. Following the reaction, the OD was recorded at 450 nm using the same microplate reader.

#### Malondialdehyde assay

Malondialdehyde (MDA) levels in tissues and cells were quantified using the lipid peroxidation assay kit (Beyotime, S0131S). Briefly, MDA reacts with thiobarbituric acid (TBA) at high temperature to form the MDA-TBA adduct, which exhibits peak absorbance at 535 nm. After the plates had cooled to room temperature, measurement of absorbance at 535 nm was performed with a microplate reader.

#### Assessment of mitochondrial membrane potential

Mitochondrial membrane potential (ΔΨm) was assessed using the JC-1 assay kit (Solarbio, M8650). GCs seeded in 48-well plates were incubated with the JC-1 working solution. Fluorescent images were captured under an inverted fluorescence microscope with dual-channel detection: J-aggregates (red fluorescence) and J-monomers (green fluorescence). The red/green fluorescence intensity ratio was calculated using ImageJ software to quantify ΔΨm changes.

#### Mitochondrial morphology analysis

Mitochondrial morphology was assessed using MitoTracker Green FM (Beyotime, C1048). Cells were stained with MitoTracker Green working solution. After PBS washing, live-cell imaging was performed using a confocal laser scanning microscope equipped with a ×60 oil objective. Images were acquired with 488-nm excitation. Mitochondrial parameters (mean branch length, mean branch diameter) were quantified using ImageJ with the mitochondrial analysis toolkit.

#### Western blotting

KGN cells and human primary GCs were lysed using RIPA buffer. We employed a BCA protein assay kit to determine protein concentrations. Samples were denatured with ×5 Laemmli buffer at high temperature for 5 min, then separated by SDS-PAGE (10%) and transferred onto PVDF membranes (Merck Millipore, USA). After washing with TBST, membranes were blocked with 5% skim milk solution for 2 h at room temperature to prevent nonspecific binding. Subsequently, membranes were incubated with diluted primary antibodies at 4°C overnight. Following three 10-min TBST washes, horse radish peroxidase (HRP)-conjugated secondary antibodies were applied for 1 h at room temperature. Protein expression was measured using an enhanced chemiluminescence kit and exposed in a chemiluminescence imaging system. The expression of the target protein was normalized by β-actin and quantified with ImageJ software. The details of primary antibodies are provided in Supplementary Table S1.

### Animal experiments

#### Animal model

Natural aging and young control models were established using 12-month-old and 12-week-old female Sprague–Dawley (SD) rats, respectively. The 12-month-old SD rat was a well-validated model for mimicking human ovarian aging [23–25]. This model was characterized by a significant decrease in anti-Müllerian hormone (AMH) levels and a marked decline in follicle count (Supplementary Figure S2). These changes were consistent with the reported features of ovarian aging in SD rats.

All animals were obtained from the Laboratory Animal Center of Chongqing Medical University. In accordance with the guidelines approved by the IACUC of the Second Affiliated Hospital of Chongqing Medical University, rats were bred and housed in a specific pathogen-free (SPF) environment. Standard conditions included a temperature of 22 ± 1°C, 60 ± 10% humidity, a 12 h light/dark cycle and *ad libitum* access to food and water. Following a 1-week acclimatization period, animals were randomly allocated into three groups (*n* ≥ 10 per group): Age, Age + PBS and Age + rhCol III. rhCol III was dissolved in sterile PBS to form a solution for administration.

Bilateral dorsal incisions ∼1 cm in length were made to expose the abdominal cavity by carefully dissecting through the subcutaneous fat and muscle layers. The ovaries, identified by their morphologically distinct mulberry-like appearance, were carefully exposed. Using an insulin microsyringe, each ovary was injected with 50 µL of either rhCol III solution (a concentration of 10 mg/mL) or an equal volume of PBS as a control. The same injection procedure was repeated 1 week after the initial administration. Rats were euthanized on Days 14 and 28 following the first injection, and both ovaries were collected from each animal for subsequent analyses.

#### Tracking of FITC-labeled rhCol III

For the FITC labeling of rhCol III, 8 mg of rhCol III was dissolved in 0.01 M PBS (pH 7.4) to prepare a 2-mg/mL solution. Two milliliters of this protein solution was transferred to an ultrafiltration unit, followed by the addition of 100 µL of FITC stock solution (2 mg/mL in dimethyl sulfoxide) (Frdbio, China). The mixture was gently mixed and incubated in the dark at 37°C for 1 h. After the reaction, an FITC scavenger was added, and the mixture was subjected to multiple rounds of centrifugation until unbound FITC was thoroughly removed.

According to the manufacturer’s guidelines, an *F*/*P* ratio between 2.5 and 6.5 was considered acceptable. The *F*/*P* ratio was calculated as follows:


$$\begin{array}{c} F/P=(A495\times C)/(A280-0.35\times A495)\\ C=(\text{MW}\times E0.1\%280)/(389\times 195) \end{array}$$

where MW denoted the molecular weight of rhCol III; A495 and A280 referred to the absorbance values of the conjugate at 495 and 280 nm, respectively; E0.1%280 referred to the absorbance value of rhCol III with a concentration of 1 mg/mL.

The FITC-labeled rhCol III was injected into the ovarian tissue. At the time points of Days 1, 3, 7, 14, 28 and 60, ovaries were harvested, fixed with 4% paraformaldehyde, and prepared as paraffin sections. After deparaffinization and rehydration, the sections were observed under the fluorescence microscope.

#### Estrous cycle assessment by vaginal smears

Following the second collagen injection, estrous cycles were monitored via daily vaginal smears. At 9:00 AM each day, vaginal secretions were collected using a moistened cotton swab, uniformly smeared onto a glass slide, and analyzed microscopically after Wright’s staining. The estrous cycle stage of rats was classified by the predominant cell types observed in the glass, as described in a previous study [26].

#### Histological analysis

In brief, rat ovarian tissues were fixed in 4% paraformaldehyde, dehydrated and embedded in paraffin. Serial sections were then cut at a thickness of 5 μm. For follicular counting, every sixth section was collected and assessed with hematoxylin and eosin (H&E). The morphology of ovarian tissue was observed with an optical microscope. The number of different follicle stages (primordial, primary, secondary, preantral and atretic follicles) was classified as previously described [27].

#### Serum hormone measurement

After rhCol III administration, blood samples were obtained from the orbital plexus of rats anesthetized with avertin. Following 1 h of coagulation at room temperature, serum was isolated by centrifugation at 3000 rpm for 10 min. Serum concentrations of estradiol (E2), AMH, luteinizing hormone (LH), and follicle-stimulating hormone (FSH) were quantified using ELISA kits. Briefly, serum specimens were added to 96-well plates pre-coated with target antigens. After 1 h incubation and subsequent addition of primary antibodies, plates were washed and incubated with HRP-conjugated secondary antibodies. Measurement of absorbance at 450 nm was performed with a microplate reader.

#### Immunohistochemistry

Ovarian tissues were fixed in 4% paraformaldehyde, embedded in paraffin and sectioned. Deparaffinization of sections was performed with xylene, followed by rehydration through a graded ethanol series. According to the immunohistochemistry kit protocol, antigen retrieval was conducted using citrate buffer, followed by blocking with 3% H_2_O_2_ and 10% goat serum in PBS. Tissue sections underwent overnight incubation with primary antibodies at 4°C. On the following day, the sections were reheated at 37°C for 30 min and subsequently washed three times with PBS.

Thereafter, the sections were exposed to a secondary antibody at 37°C for 30 min, followed by staining with diaminobenzidine (DAB) for histological visualization. For each section, three non-overlapping fields were randomly selected under ×100 magnification for subsequent analysis. All acquired images were quantitatively evaluated using ImageJ software. The information on primary antibodies was listed in Supplementary Table S1.

#### Immunofluorescence staining

Paraffin sections were deparaffinized and rehydrated using standard protocols. Subsequently, 10% goat serum was used to block the sections for 1 h to prevent nonspecific binding. Thereafter, the sections were incubated with diluted primary antibodies at 4°C overnight. After washing three times with PBS, the sections were incubated with a fluorescently labeled secondary antibody for 1 h at 37°C in the dark. Nuclei were counterstained with DAPI solution. Finally, images were captured using a fluorescence microscope (Nikon). The acquired images were analyzed with ImageJ to quantify the mean fluorescence intensity.

#### TUNEL analysis

As previously described [28], paraffin-embedded sections were deparaffinized and rehydrated. The sections were then covered with TUNEL reaction mixture and incubated at 37°C for 2 h in the dark. The section was observed using a fluorescence microscope (Nikon). Apoptotic cells were identified by a positive red fluorescence signal. Apoptosis was quantified based on the fluorescence intensity using ImageJ.

#### Transmission electron microscopy

Following primary fixation with 2.5% glutaraldehyde at 4°C overnight, rat ovarian tissues were post-fixed with 1% osmium tetroxide. Embedding was performed in EPON-812 resin subsequent to sample dehydration. Ultrathin sections, collected on copper grids, underwent double-staining using uranyl acetate and lead citrate. Images were acquired using a JEM-1400FLASH TEM (JEOL, Japan) at 80 kV accelerating voltage.

#### Scanning electron microscope

Tissue specimens were immersed in 3% glutaraldehyde at 4°C for 24 h, washed thrice with ultrapure water (3 min/wash), and post-fixed in 1% osmium tetroxide. After repeated washes, dehydration was performed in an ethanol gradient. Critical-point dried specimens were sputter-coated with gold film and observed using a JSM-IT700HR SEM (JEOL, Japan).

#### Collagen fiber and pore quantification

SEM images were quantified by means of the Diameter J plugin (v2.0) for ImageJ. Briefly, images were binarized via Otsu thresholding (black pixels = background, white = fibers). Fiber diameter was analyzed by fitting Gaussian curves to radius distributions and extracting mean values. Pores were identified as contiguous black pixel clusters. Mean pore area was derived from all detected pores. Fiber density was quantified as pore percentage: (total black pixels/total pixels) × 100%.

#### Fiber orientation analysis

Fiber orientation was assessed in 3–5 randomly selected SEM fields per sample. Fiber angles (0–180°) relative to the Cartesian coordinate system were measured by tracing fiber endpoints. Pixel intensity distribution was quantified at specific angles. Mean orientation and circular histograms were generated using Origin software (version 2022), and data were visualized on half-polar plots (0–180°).

### Bioinformatics analysis

#### RNA sequencing

Total RNA was isolated with TRIzol Reagent, and its integrity was assessed on an Agilent 2100 Bioanalyzer (Agilent Technologies). Only samples meeting the quality criterion (RIN > 8) were processed for library preparation.

Library sequencing was performed on the Illumina Nova Seq 6000 platform (Illumina, San Diego, CA, USA) with 150-bp paired-end reads according to the manufacturer’s protocol. We utilized DESeq2 for differential gene expression analysis (v1.38.3). Genes meeting the threshold of adjusted *P* values (*P* adj) < 0.05 and |log2 fold change| >1 were defined as differentially expressed genes (DEGs).

#### GC ScRNA-seq data collection and preparation

The scRNA-seq data of GCs, GSE255690 were obtained from the Gene Expression Omnibus (GEO) database. Cells expressing between 300 and 5000 genes were retained for further analysis, while those exhibiting high mitochondrial gene contamination were excluded. Batch effect correction was performed using the Harmony R package. Cell clusters were identified via the FindClusters function in Seurat (v 4.0.0) and annotated based on well-established marker genes collected from the literature. DEGs across GC subpopulations were identified using the FindMarkers function. Venn diagrams were employed to compare DEGs between two datasets. Enrichment analyses for Gene Ontology (GO) terms and Kyoto Encyclopedia of Genes and Genomes (KEGG) pathways were conducted based on the hypergeometric distribution algorithm. Significantly enriched terms were visualized using bar plots generated with R software (v 3.2.0).

### Statistical analysis

Statistical analyses are performed using GraphPad Prism software (version 9.5). One-way analysis of variance (ANOVA) was applied to compare variables among groups. When significant differences are detected (*P* < 0.05), Fisher’s least significant difference (LSD) test was conducted for multiple comparisons with confirmed homogeneity of variances. Data are shown as mean ± standard deviation. Statistical significance is presented as *P < 0.05, **P < 0.01 and ***P < 0.001. All graphical representations are generated using GraphPad Prism software (version 9.5).

## Results

### The expression of Col III in ovarian tissues of humans and rats

**Figure 1:**
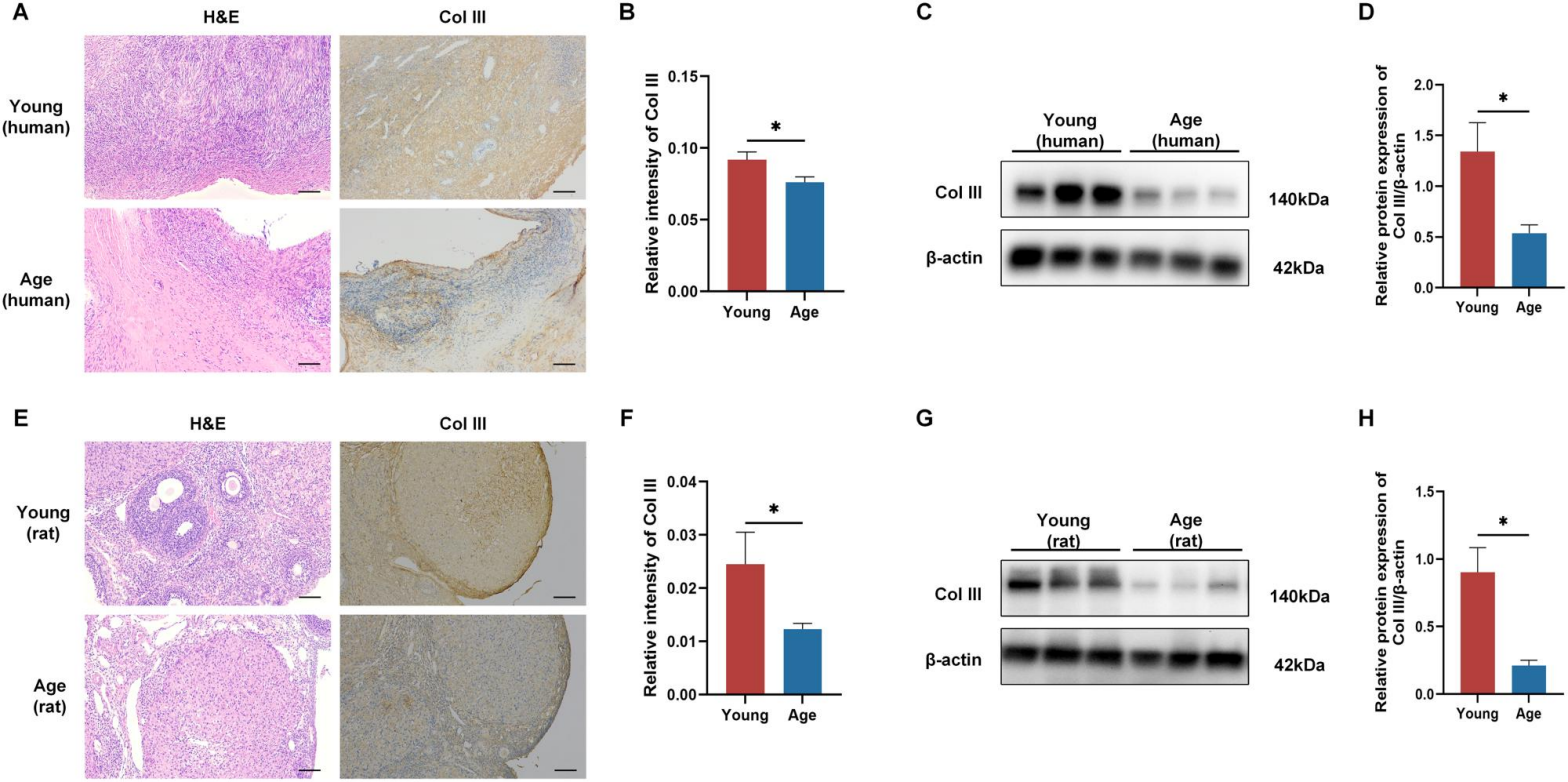
The expression of Col III in humans and the rat model. (**A**) The images of H&E staining and Col III in human ovarian tissues (*n* = 10). Scale bar = 100 µm. (**B**) Statistical analysis of Col III expression. (**C** and **D**) The expression of Col III was detected by western blotting (*n* = 10). (**E** and **F**) H&E staining and immunohistochemistry analyses of Col III (*n* = 10). Scale bar = 100 µm. (**G** and **H**) The expression of Col III was explored by western blotting (*n* = 10) (*P < 0.05, **P < 0.01, ***P < 0.001).

We investigated the expression pattern of Col III in the ovarian tissues of humans and rats. Compared with the Young group, the Col III expression in human ovarian tissues was significantly decreased in the Age group (Figure 1A and B), a finding further assessed by western blotting (Figure 1C and D). Similarly, in the rat model, Col III expression was significantly lower in the Age group than in the Young group (Figure 1E and F), which was also validated by Western blot analysis (Figure 1G and H). These results suggested that a deficiency of Col III expression may contribute to the decline in ovarian function.

### rhCol III possesses a distinctive structure as a biomaterial

**Figure 2:**
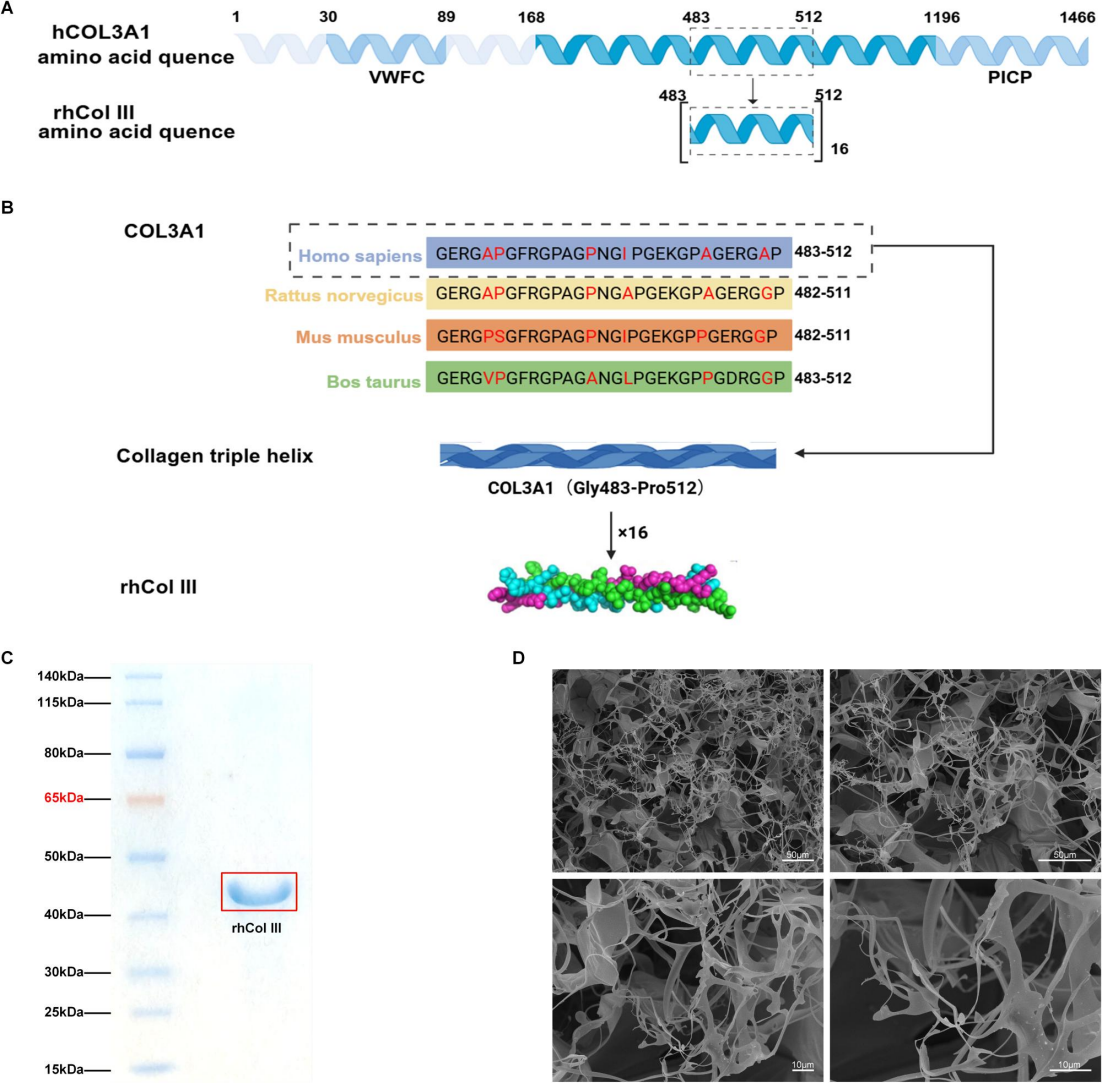
rhCol III has a distinctive structure as a biomaterial. (**A**) The amino acid sequence of rhCol III. (**B**) The assembly process of rhCol III. (**C**) the molecular weight of rhCol III was determined by SDS-PAGE analysis. (**D**) SEM image of rhCol III. Scale bar =50 or 10 μm.

As shown in our previous study, hCOL3A1 was composed of 1466 amino acids. The specific repeating structure of hCOL3A1 483-512 amino acid segment was recorded in the Protein Data Bank (No: 6A0C, 6A0A) (Figure 2A). The amino acid sequence of rhCol III was derived exclusively from the human *COL3A1* gene (Figure 2B). The human origin minimizes the risk of potential immunogenicity associated with non-human sequences, such as those from murine or porcine sources. Given the high sequence homology between rat and human *COL3A1*, SD rats were selected as the animal model for subsequent experiments. The characteristic triple-helical structure of rhCol III was formed by 16 tandem repeats of the Gly–Lys–Glu and Gly–Glu–Arg motifs, which served as the structural foundation for its biological function (Figure 2B). The apparent molecular mass of rhCol III was estimated to be 43 kDa based on the SDS-PAGE analysis (Figure 2C). Furthermore, examination under SEM revealed that the material self-assembled into a 3D network of fine and flexible fibers, with pore diameters ranging between 20 and 30 μm (Figure 2D).

### rhCol III ameliorated ovarian function in natural aging rat models

**Figure 3:**
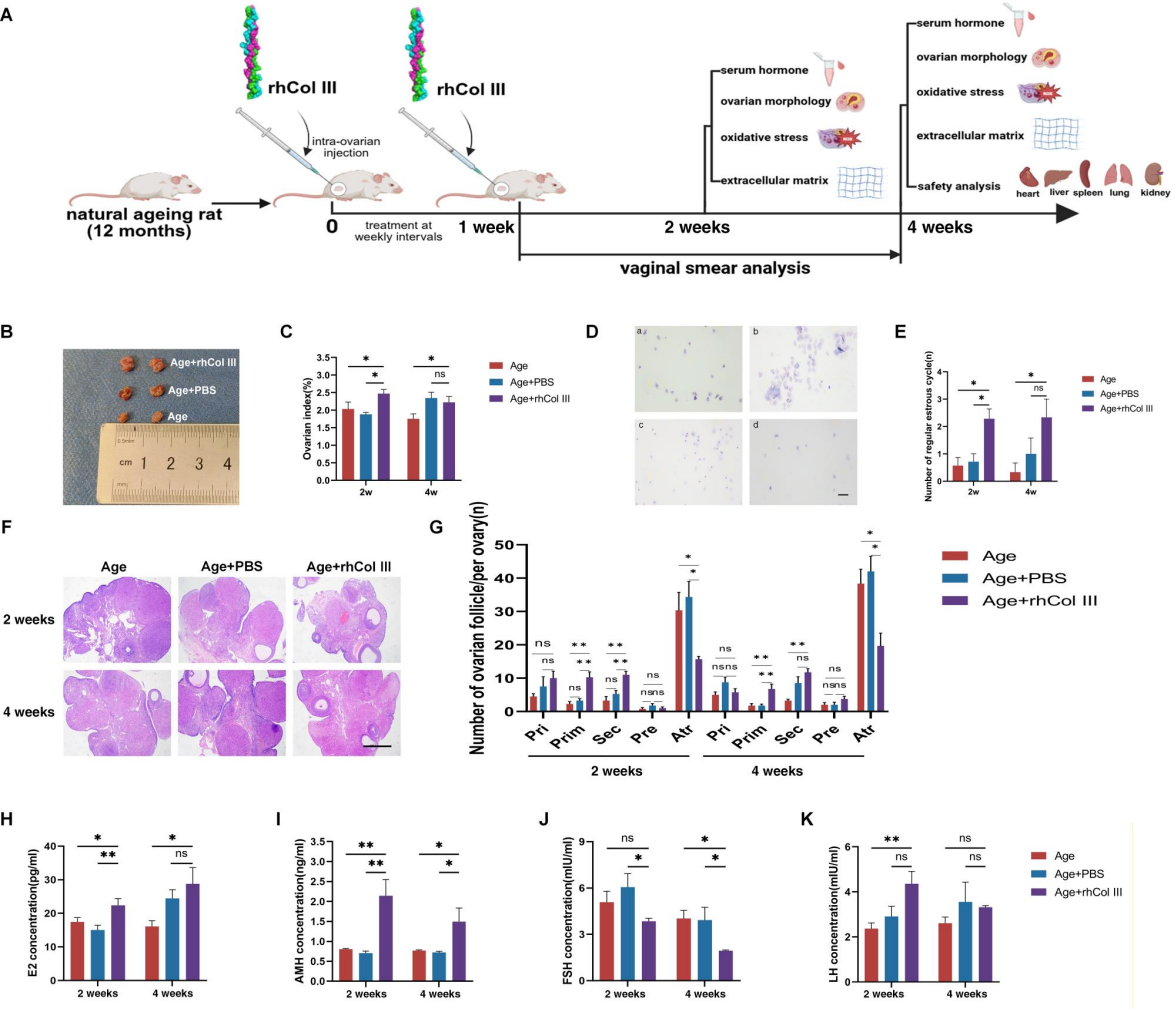
rhCol III improved ovarian function in naturally aging rat. (**A**) The procedure of animal experiments. (**B**) The images of the ovary from the age, PBS and rhCol III group. (**C**) The ovary weight index was measured at 2 and 4 weeks after rhCol III injection (*n* = 5). (**D**) Images of vaginal smear indicating different stages of the estrous cycle. Scale bar = 100 µm. a: proestrus, b: estrus, c: metestrus, d: diestrus. (**E**) The number of regular estrous cycle (*n* = 5). (**F**) Ovarian morphology was revealed by H&E staining. Scale bar = 100 µm. (**G**) Analysis of follicle number at different stages in ovaries (*n* = 5). Pri: Primordial follicles, Prim: Primary follicles, Sec: Secondary follicles, Pre: Preovulatory follicle, Atr: Atretic follicles. (**H**–**K**) The serum level of E2, AMH, FSH and LH were measured by ELISA analysis at 2 and 4 weeks after rhCol III treatment (*n* = 5). (*P < 0.05, **P < 0.01, ***P < 0.001).

To evaluate the efficacy of rhCol III in aging rats, the estrous cycle, ovarian index, sex hormone levels and longitudinal evaluation of ovarian morphology were performed (Figure 3A). The ovarian index was determined based on the ratio of ovarian weight/body weight. At 2 and 4 weeks after rhCol III injection, the ovarian index was obviously increased in the rhCol III group compared with the Age group, while no statistical difference was observed between the Age and PBS group (Figure 3B and C). Vaginal smears were used to assess the recovery of the estrous cycle (Figure 3D). A regular 4–5-day estrous cycle was considered normal. The Age group had disrupted estrous cycles, and PBS group was similar to that. The rhCol III group exhibited significant recovery at the 2- and 4-week time points (Figure 3E). Furthermore, ovarian morphology and follicle count were observed via H&E staining (Figure 3F). Compared with the Age and PBS group, the number of growing healthy follicles, including primary and secondary follicles, was statistically increased, and the number of atretic follicles was obviously decreased in the rhCol III group (Figure 3G). In addition, no statistical differences in serum levels of E2, AMH, LH and FSH were observed between the Age and PBS groups. The level of AMH and E2 was significantly increased in the rhCol III group (Figure 3H and I) compared to the Age group. The level of FSH was reduced in the rhCol III group (Figure 3J). Interestingly, LH concentration was observed to increase in the rhCol III group at 2 weeks compared to the Age group, with no significant difference at 4 weeks (Figure 3K).

### rhCol III alleviated oxidative stress, promoted angiogenesis and reduced GC apoptosis at the tissue level

**Figure 4:**
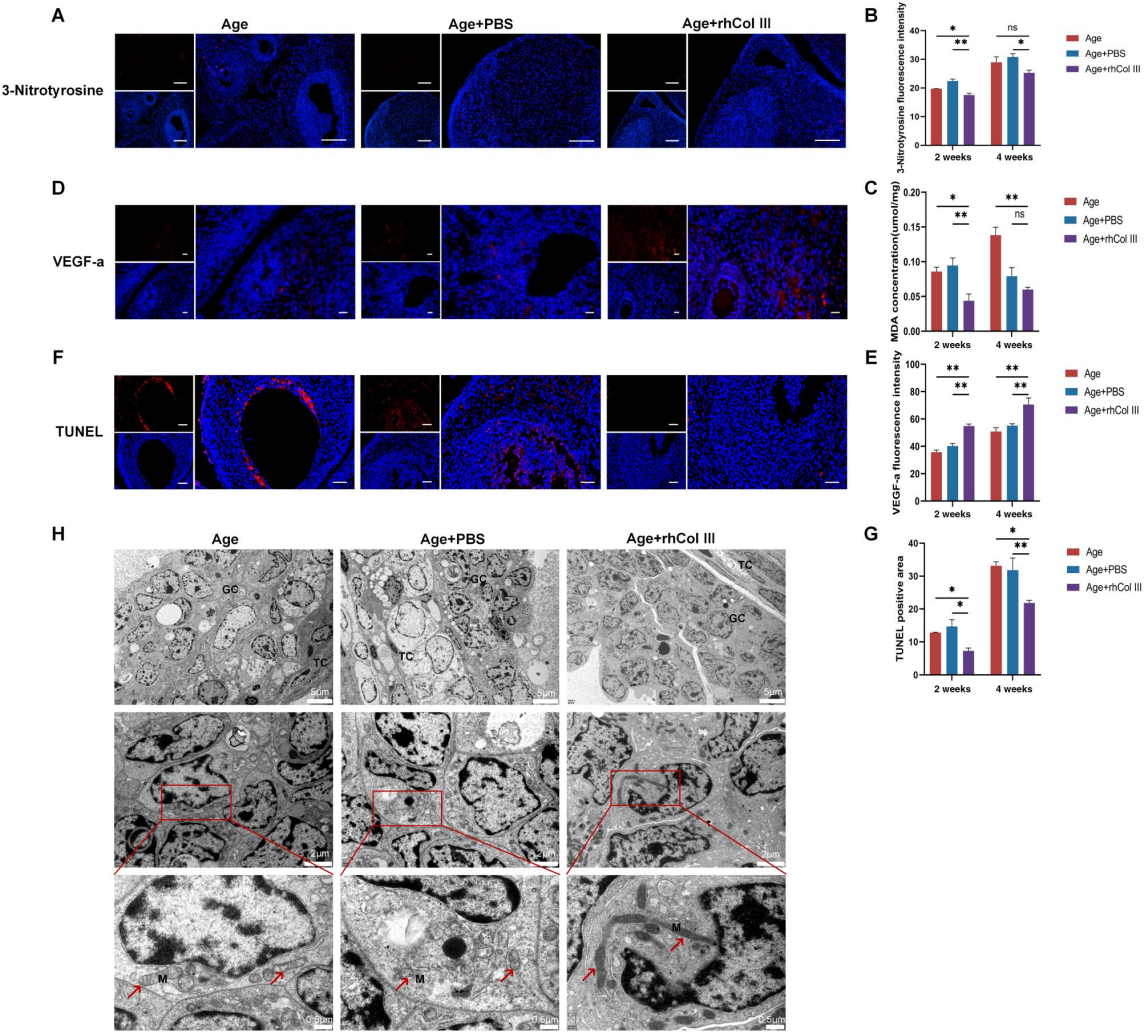
The protective effect of rhCol III on the ovary at the tissue level. (**A**) Fluorescence images of the ovary stained for 3-NTY (*n* = 5). Scale bar = 100 µm. (**B**) Quantitative analysis of 3-NTY fluorescence density. (**C**) The level of MDA in the ovary (*n* = 5). (**D**) Images of the ovary stained for VEGF-A. Scale bar = 25 µm. (**E**) Analysis of VEGF-A mean fluorescence density (*n* = 5). (**F**) The TUNEL staining was conducted to visualize GC apoptosis (*n* = 5). Red fluorescence represented apoptotic GCs. Blue fluorescence represented the nucleus. Scale bar = 50 µm. (**G**) Quantitative analysis of TUNEL. (**H**) TEM images of ovarian GC layers among three groups. Scale bar = 5  or 2 µm. GC: granulosa cell; M: mitochondria; TC: Theca cell (*P < 0.05, **P < 0.01, ***P < 0.001).

Previous experiments showed that rhCol III could restore the growth of follicles and recover the serum level of sex hormones. Then, we investigated the effects of rhCol III on ovarian tissue (cortex and medulla). MDA and 3-nitrotyrosine (3-NTY) are recognized as markers of oxidative stress, which are also closely associated with ovarian aging. The level of 3-NTY showed no statistical difference between the Age group and the PBS group, while a downward trend of 3-NTY level was observed in the rhCol III group at two time points (Figure 4A and B). Additionally, the MDA level was assessed in both the Age group and PBS group, with no statistical difference. However, the level of MDA exhibited a significant decrease in the rhCol III group after 2 and 4 weeks of treatment (Figure 4C).

The ovarian medulla is mainly composed of blood vessels and lymphatic vessels. Vascular endothelial growth factor A (VEGF-a) is a marker of increased angiogenesis. The level of VEGF-a showed a significant upward trend in the rhCol III group at two time points (Figure 4D and E). The cortex consists of follicles, which are composed of oocytes and GCs. TUNEL staining was conducted to detect the apoptosis of GCs (Figure 4F). Red fluorescence represented apoptotic cells, which were situated in the GC layer. The apoptotic cells were reduced in the rhCol III group, compared with the Age and PBS group (Figure 4G).

Finally, to observe the microscopic changes of GCs, TEM was conducted. Notably, the Age and PBS groups exhibited more abnormal mitochondria, characterized by damaged cristae and round morphology. Treatment with rhCol III attenuated these damages and restored the mitochondrial morphology with obvious cristae (Figure 4H). These results suggested that rhCol III could inhibit GC apoptosis and rescue mitochondrial morphology, thereby contributing to the growth of healthy follicles.

### rhCol III was deposited in the ovary and remodeled the extracellular matrix

**Figure 5:**
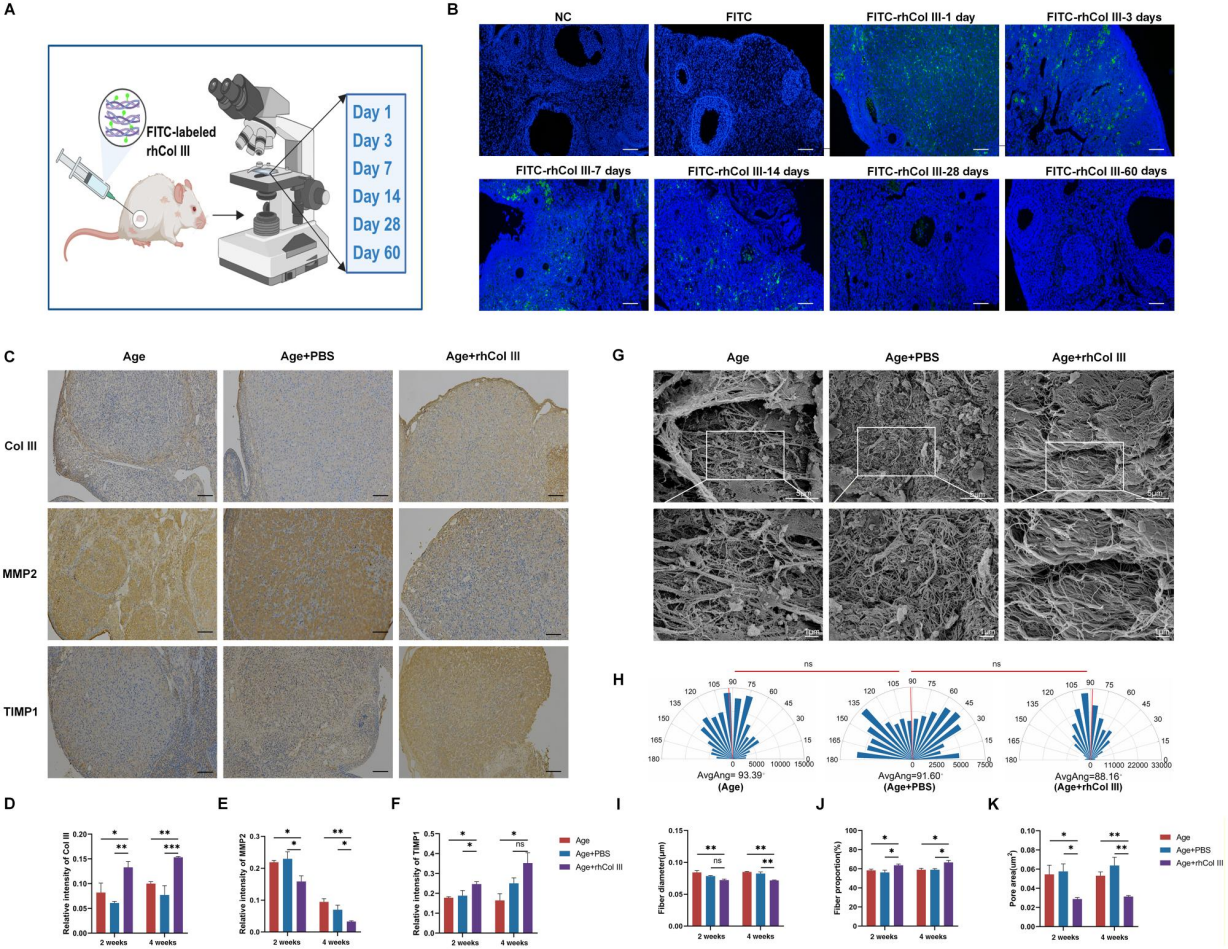
The localization of labeled rhCol III and evidence of rhCol III remodeling ECM. (**A**) A schematic diagram of rhCol III observation. (**B**) Tracking of labeled rhCol III in the ovaries at different time points (Days 1, 3, 7, 14, 28 and 60). Scale bar =100 µm. (**C**) Immunohistochemical staining of Col III, MMP2 and TIMP1. Scale bar =100 µm. (**D**–**F**) Statistical analysis of Col III, MMP2 and TIMP1 (*n* = 4). (**G**) SEM images of age, PBS and rhCol III group. Scale bar =5 or 1 µm. (**H**) Orientation analysis of collagen fiber (*n* = 3). (**I**) Statistical analysis of fiber diameter (*n* = 3). (**J**) Statistical analysis of fiber proportion (*n* = 3). (**K**) Quantitative analysis of pore area (*n* = 3) (*P < 0.05, **P < 0.01, ***P < 0.001).

For *in vivo* tracking, rhCol III was conjugated with FITC. The calculated *F*/*P* ratio of 3.03 indicated adequate labeling of rhCol III. The tissues injected with FITC-labeled rhCol III were observed under a fluorescence microscope (Figure 5A). On Day 1 after injection, the fluorescent signal was diffused throughout the ovarian cortex. From Day 1 to 14, rhCol III was observed throughout the whole ovarian tissue. Fluorescence intensity began to decrease on Day 28 and was nearly undetectable on Day 60 (Figure 5B).

The ECM provides structural support and biochemical signals for follicular growth and development. The biomechanical properties determined by ECM molecules are crucial for follicle activation. ECM components were analyzed by immunohistochemistry at two time points including 2-week and 4-week (Figure 5C). No significant difference was observed in Col III between the Age and PBS group at the 2- or 4-week time point. Compared with the Age and PBS group, the expression of Col III was significantly increased in the ovarian cortex of animals in the rhCol III group at 2 weeks (Figure 5D). In the next time point, Col III exhibited an upward trend. TIMP1 and MMP2 are key regulators of ECM remodeling. The expression of TIMP1 and MMP2 was similar between the Age and PBS groups. However, the expression of MMP2 exhibited a decremental trend in the rhCol III group at 2- and 4-week time points (Figure 5E). In contrast, TIMP1 expression was significantly increased in the rhCol III group at 2 and 4 weeks (Figure 5F).

The endogenous Col III expression was promoted by rhCol III. To further explore the influence of rhCol III on collagen fiber morphology, SEM was performed. Main features of the collagen fibers were captured at **×**5000 and **×**10 000 magnifications. Compared to the Age group, the PBS group showed no statistically significant change in collagen fiber morphology. Collagen fibers in two groups exhibited a disordered and irregular pattern, while fibers in the rhCol III group were regularly arranged, with an aligned orientation (Figure 5G). The characteristics of collagen fibers including their diameter, density and orientation, were quantified to analyze the effect of rhCol III on ECM structure. Though the mean fiber orientation of the rhCol III group was smaller than that of the other groups, the difference was not statistically significant (Figure 5H). Fibers in the Age and PBS groups were formed into bundles with a thicker diameter. The rhCol III group showed a different organization of ECM, characterized by thinner fibers (Figure 5I). Fiber proportion and pore area were analyzed to evaluate the tightness of the tissue network. Lower fiber proportion and larger pore areas were observed in the Age and PBS group, indicating a loose tissue network. In contrast, the rhCol III group exhibited reduced pore areas and higher fiber proportion, forming a tight network (Figure 5J and K). These results implied that treatment with rhCol III could restore aged-related structural disorganization of the ECM, contributing to the structural integrity of the follicular scaffold. In addition to its efficacy in protecting ovarian function and ECM structure, rhCol III exhibited a favorable biosafety profile (Supplementary Figure S3).

### rhCol III promoted proliferation, rescued senescence, improved mitochondrial function and reduced oxidative stress in aged GCs

**Figure 6:**
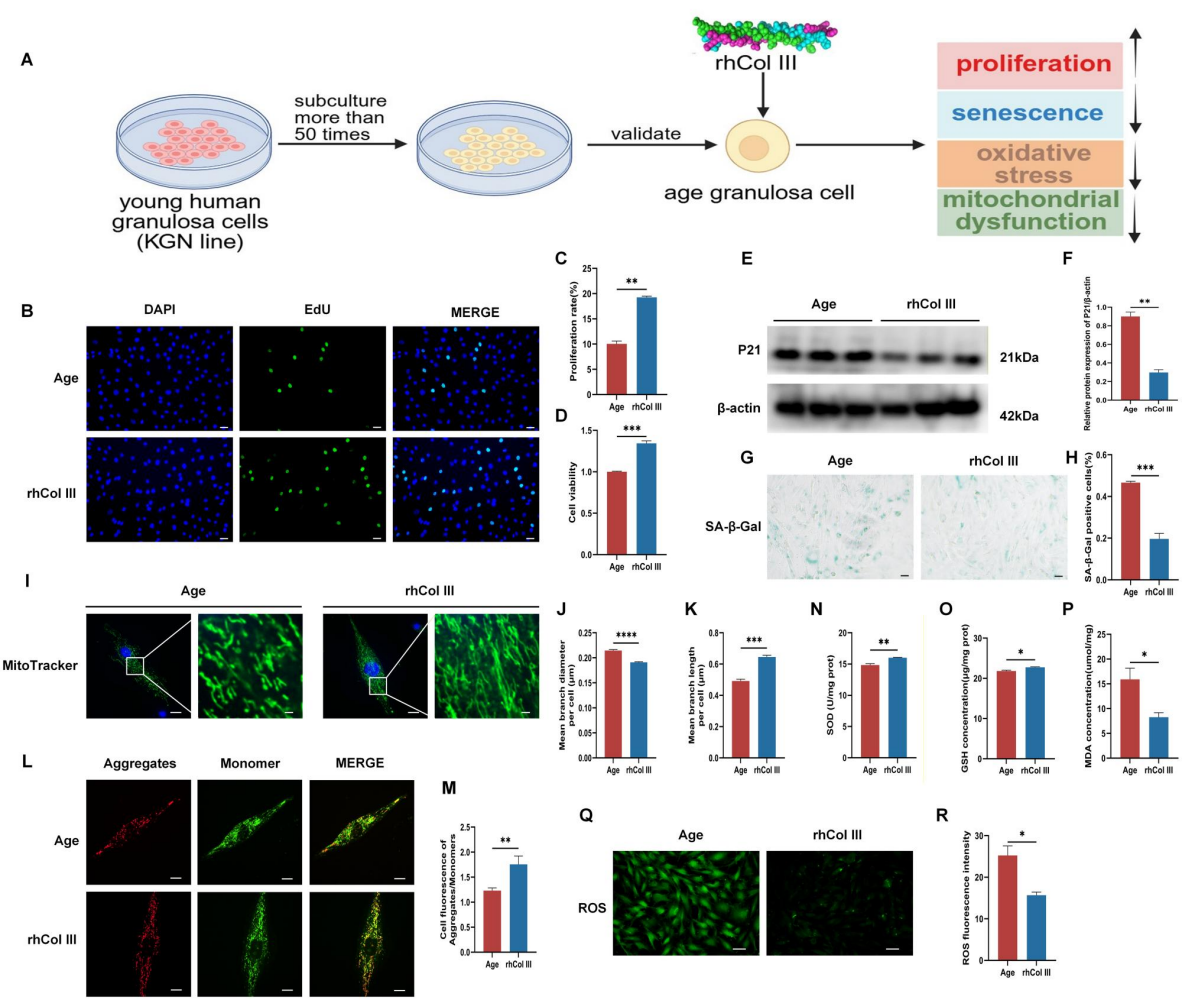
rhCol III promoted GC proliferation, rescued cellular senescence and improved mitochondrial function. Data were representative of three independent experiments. (**A**) Schematic procedure of cell experiments. (**B**) Cell proliferation was assessed by EdU assay. Green fluorescence represented proliferating cells. Blue fluorescence represented the nucleus. Scale bar = 50 µm. (**C**) Statistical analysis of EdU assay. (**D**) Cell proliferation was analyzed by CCK-8 assay. (**E**) The expression of P21 was determined by western blotting. (**F**) Statistical analysis of P21 expression. (**G**) Cellular senescence was revealed by SA-β-gal staining. Scale bar = 50 µm. (**H**) Statistical analysis of senescent cell. (**I**) Mitochondrial morphology was labeled using a MitoTracker probe. The morphology was labeled by green fluorescence. Scale bar = 20 µm. (**J** and **K**) Quantitative analysis of mitochondrial morphology. (**L**) Images of JC-1 staining in age and rhCol III group. Red fluorescence represented aggregates. Green fluorescence represented monomers. Scale bar = 20 µm. (**M**) Quantitative analysis of mitochondrial membrane potential (ΔΨm). (**N**–**P**) The level of SOD, GSH and MDA were measured by kit. (**Q**) Intracellular ROS was detected by DCFH-DA probe. Scale bar = 50 µm. (**R**) Statistical analysis of ROS accumulation (*P < 0.05, **P < 0.01, ***P < 0.001).

The KGN line was employed to explore the effect of rhCol III on human GCs *in vitro* (Figure 6A). An aged GC model was established by >50 successive passages. The expression of P21, a senescence marker, exhibited an upward trend in the Age group compared to the Young group (Supplementary Figure S4A and B). Cellular senescence was also confirmed by SA-β-gal staining (Supplementary Figure S4C and D). Aged GCs were treated with 8 mg/mL of rhCol III for 24 h. Then, EdU proliferation analysis was performed, which showed a statistical increase in the number of proliferating cells in the rhCol III group compared to the Age group (Figure 6B and C). A Similar result was observed by CCK-8 assay (Figure 6D). Additionally, the expression of P21 was statistically decreased in the rhCol III group (Figure 6E and F). SA-β-gal staining was also conducted to examine cellular senescence. SA-β-gal staining showed that the number of positive cells was statistically increased in the Age group, while reduced in the rhCol III group (Figure 6G and H).

Based on the efficacy of rhCol III for rescuing senescence in aged GCs, we next investigated the effect of rhCol III on the organelles of aged GCs. Specifically, mitochondrial dysfunction was shown to be associated with ovarian aging. TEM revealed obvious mitochondrial damage in the GC layer of aged rats (Figure 4H). Thus, we explored the changes of mitochondrial function of GCs. MitoTracker probes were used to label mitochondrial structure in live cells. Abnormal mitochondrial morphology, including fragmentation and swelling, was observed in aged GCs, and rhCol III rescued these changes (Figure 6I). Further quantitative analysis of mitochondrial structure indicated that mitochondria were significantly shorter and thicker in the aged GCs than in the rhCol III group, suggesting that rhCol III could restore the mitochondrial morphology (Figure 6J and K).

JC-1 staining was employed to measure mitochondrial membrane potential. More monomers were found in age GCs, while rhCol III reduced the number of monomers and promoted aggregate formation (Figure 6L). These changes were confirmed by the ratio of aggregates/monomers (Figure 6M). Mitochondrial dysfunction leads to excessive ROS production and increased oxidative stress. Oxidative stress markers including MDA, SOD and GSH were measured. Antioxidant index, involving SOD and GSH, was decreased in aged GCs, and rhCol III significantly promoted their production (Figure 6N and O). MDA, as an oxidative stress product, was accumulated in aged GCs and was reduced by rhCol III (Figure 6P). Additionally, the DCFH-DA probe detected intracellular ROS accumulation, which was elevated in aged GCs but significantly reduced by rhCol III (Figure 6Q and R). These results suggested that rhCol III could rescue mitochondrial function and improve the aged state in GCs.

### Bioinformatic analysis implicated ITGA2-PI3K/Akt signaling in rhCol III-mediated improvements

**Figure 7:**
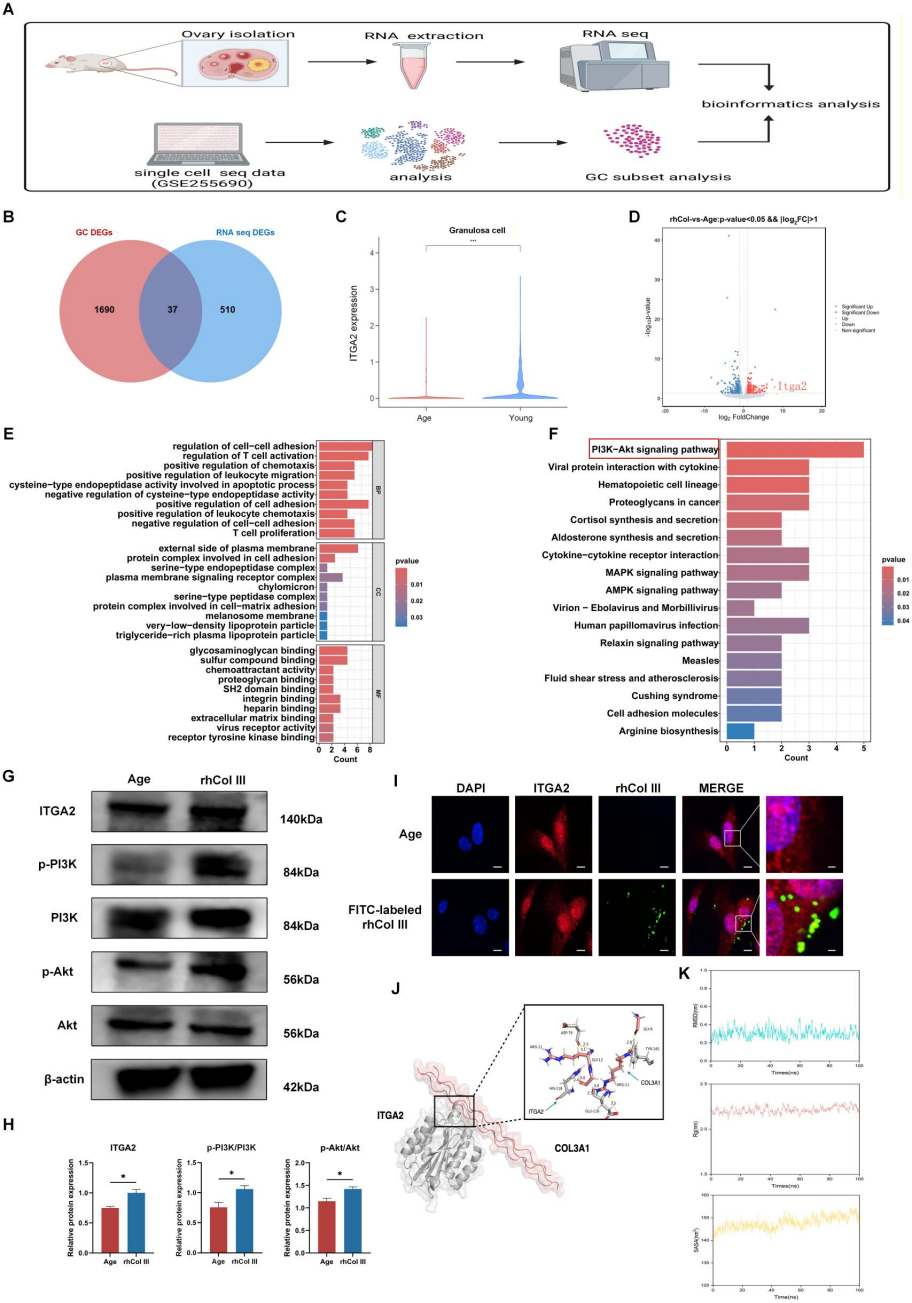
rhCol III interacted with ITGA2 and activated the PI3K/Akt pathway. (**A**) Schematic diagram of the analysis. (**B**) Venn analysis showing DEGs between RNA-seq data and GC single-cell seq data. (**C**) The differential expression of ITGA2 in young and aged groups in GC single-cell seq. (**D**) Volcano plot showing DEGs in the aged group compared to the rhCol III group. (**E**) GO analysis of DEGs. (**F**) KEGG analysis of DEGs. (**G**) The activity of ITGA2, p-PI3K, PI3K, p-Akt and Akt was assessed by western blotting. (**H**) Statistical analysis of ITGA2, p-PI3K/PI3K and p-Akt/Akt. (**I**) The binding between rhCol III and ITGA2 was confirmed by immunofluorescence co-localization. Red fluorescence represented ITGA2. Green fluorescence represented FITC-labeled rhCol III. Blue fluorescence represented cell nucleus. Scale bar = 20 or 10 µm. (**J**) Conformational dynamics and interaction landscape of the rhCol III-ITGA2 complex were characterized through molecular dynamics simulations, revealing key binding residues. (**K**) Quantitative assessment of the stability of rhCol III-ITGA2 complex during 100-ns MD simulations. Key metrics included RMSD, Rg and SASA. (*P < 0.05, **P < 0.01, ***P < 0.001).

To explore how rhCol III improves the function of GC, RNA sequencing of ovarian tissues was performed between the Age and rhCol III groups. Besides, a single-cell sequence dataset including young and aged GCs was obtained from the GEO database (Figure 7A). After processing the raw data, DEGs were identified. Venn analysis was conducted to identify 37 DEGs in both datasets (Figure 7B). Bioinformatics analysis indicated that integrin α2 (ITGA2) was downregulated in aged ovaries but restored by rhCol III (Figure 7C and D). Notably, we examined the expression level of ITGA2 in clinical samples and found that its expression was significantly higher in GCs from young women than in GCs from aged women (Supplementary Figure S5A and B). Correlation analysis indicated that ITGA2 expression was positively correlated with AMH levels and antral follicle counting (AFC) (Supplementary Figure S5C and D), suggesting that ITGA2 may serve as a critical role in the maintenance of ovarian function. GO and KEGG analyses were performed to identify potential pathways. DEGs were enriched in functions related to cell–cell adhesion and integrin binding, indicating interactions between rhCol III and cells (Figure 7E). KEGG analysis revealed that activation of the PI3K/Akt signaling pathway was involved in the effect of rhCol III (Figure 7F).

Based on the results of bioinformatic analysis, we preliminarily explored the involved signal pathway through which rhCol III improved mitochondrial function in GCs. Western blotting was used to assess the activity of ITGA2, Akt, and PI3K (Figure 7G). The expression of ITGA2, phosphorylated Akt and phosphorylated PI3K was significantly increased in the rhCol III group (Figure 7H). Immunofluorescence co-localization analysis was conducted to demonstrate the binding between rhCol III and ITGA2. FITC-labeled rhCol III was predominantly localized on the plasma membrane of GCs, which was similar to the distribution of ITGA2 (Figure 7I). Besides, molecular docking analysis was performed to confirm the binding activity between rhCol III and ITGA2. Structural visualization revealed that specific interactions at the amino acid level contributed to the stability of the rhCol III-ITGA2 complex (Figure 7J). Quantitative metrics supported complex stability over a 100-ns simulation period (Figure 7K). The root mean square deviation (RMSD) fluctuated within 0.3 nm indicating the conformational stability of the complex. Additionally, the radius of gyration (Rg) revealed a compact interface between rhCol III and ITGA2. The stability of the complex was also confirmed by the solvent accessible surface area (SASA). These results implied that rhCol III may bind to ITGA2 on GCs and activate the PI3K/Akt signaling pathway.

### rhCol III restored the mitochondrial function of aged GCs via ITGA2 signaling

**Figure 8:**
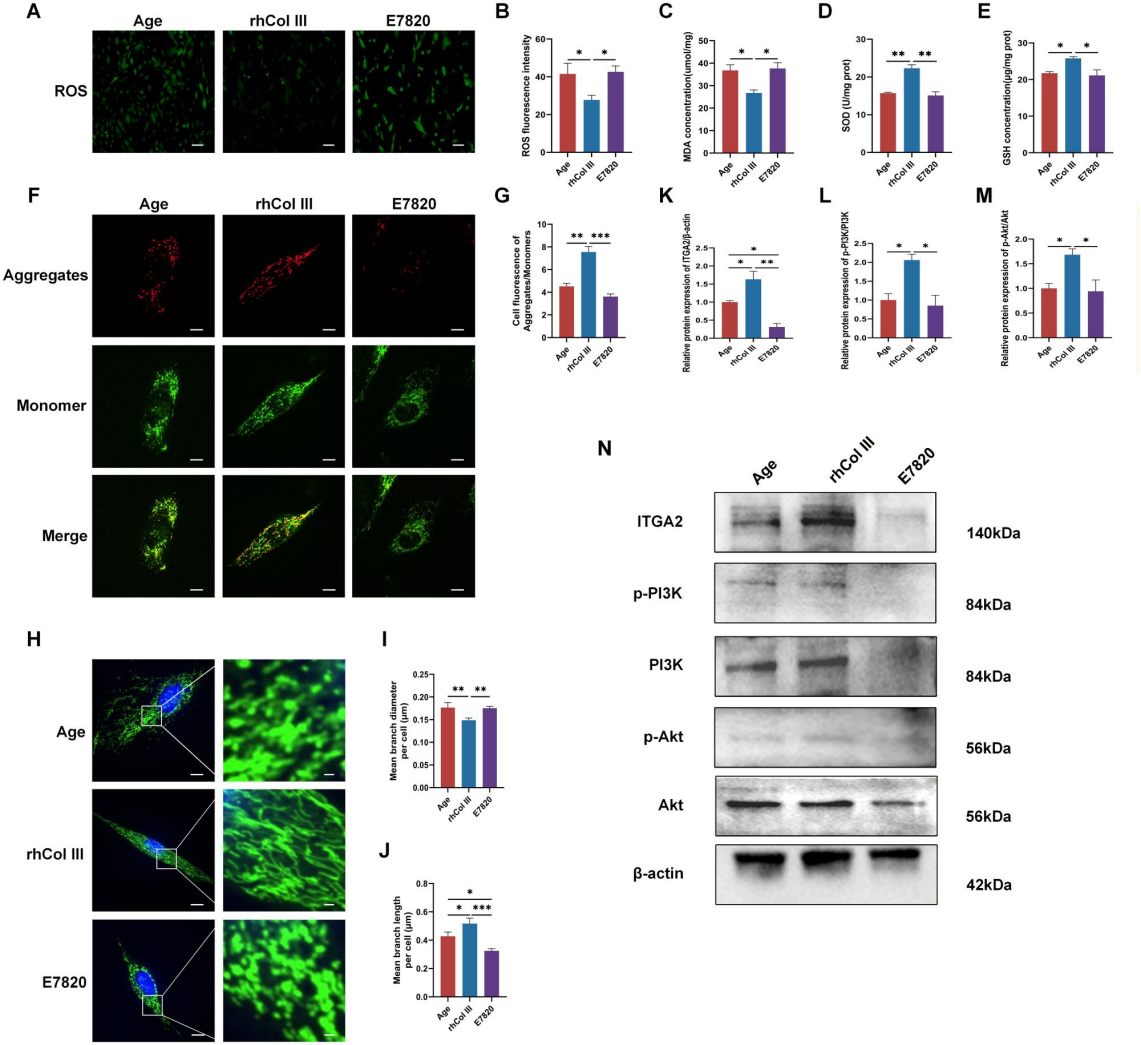
rhCol III Improved cellular mitochondrial function and rescued oxidative stress by binding to ITGA2. Data were representative of three independent experiments. (**A**) ROS accumulation was assessed by DCFH-DA probe among three groups. Scale bar =20 µm. (**B**) Statistical analysis of ROS. (**C**–**E**) The intracellular levels of MDA, SOD and GSH were measured. (**F**) JC-1 staining was employed to detect mitochondrial membrane potential. Scale bar =20 µm. (**G**) Statistical analysis of mitochondrial membrane potential (ΔΨm). (**H**) Mitochondrial morphology of three groups. Scale bar =20 µm. (**I** and **J**) Quantitative assessment of mitochondrial morphology including length and diameter. (**K**–**M**) Statistical analysis of ITGA2, p-Akt/Akt, and p-PI3K/PI3K. (**N**) Western blotting showing the activity of ITGA2, p-Akt, Akt, p-PI3K and PI3K (*P < 0.05, **P < 0.01, ***P < 0.001).

To validate whether rhCol III rescues mitochondrial function through ITGA2, the specific ITGA2 inhibitor E7820 was used to suppress the expression of ITGA2. Oxidative stress was assessed based on ROS detection and MDA level. The results showed that E7820 prevented rhCol III from rescuing ROS accumulation and reducing MDA concentration (Figure 8A–C). Antioxidants, including SOD and GSH, were increased in the rhCol III group but not in the E7820 group, suggesting that rhCol III regulated oxidative stress by interacting with membrane ITGA2 receptors (Figure 8D and E). JC-1 and MitoTracker staining were used to assess mitochondrial morphology and function. JC-1 staining indicated that E7820 increased monomer formation and reduced aggregate formation by reducing ITGA2 levels (Figure 8F and G). MitoTracker probes showed that rhCol III alone improved mitochondrial morphology, while E7820 inhibited this effect (Figure 8H–J). Finally, the activity of the PI3K/Akt signal pathway was demonstrated by western blotting (Figure 8N). Except for reducing ITGA2 expression, E7820 inhibited the expression of phosphorylated Akt and PI3K (Figure 8K–M). Collectively, these results suggested that rhCol III improved mitochondrial function in aged GCs by interacting with ITGA2 and activating the PI3K/Akt signal pathway.

## Discussion

Ovarian aging is primarily characterized by depletion of ovarian follicles. Current therapeutic strategies for ovarian aging remain limited. For example, hormone replacement therapy is accompanied by an increased risk of cancer despite alleviating the symptoms of estrogen-deficiency [29, 30]. In this study, we first demonstrated that a deficiency of Col III was closely associated with ovarian function decline. Treatment with rhCol III effectively restored ovarian function and attenuated ovarian damage in aging rats by reconstructing the ECM scaffold, which was essential for follicular growth. Furthermore, we discovered that rhCol III interacted with ITGA2 receptors on GCs, thereby promoting their proliferation, mitigating oxidative stress, and ameliorating cellular senescence. These findings provide novel insights into potential targets for follicular development and propose a new therapeutic perspective for ovarian aging.

Ovarian endocrine function and oocyte release fundamentally rely on follicles. Collagen, a major component of ECM, plays a critical role in maintaining the structural and biochemical microenvironment essential for follicle production [31]. Several studies have shown that genetic deficiencies in collagen-related genes were associated with impaired follicular development [32, 33]. Furthermore, collagen-based hydrogels were shown to enhance estrogen secretion of GCs and promote oocyte maturation *in vitro* [34]. The biological effects of different types of collagen are likely associated with their distinct localization in the ovaries [35]. Col I primarily exists in the ovarian stroma [36], where its expression is regulated by transforming growth factor beta 1 (TGF-β1) signaling [37]. The expression of Col I was found to significantly decrease during corpus luteum formation [38]. In contrast, Col IV is mainly distributed in the basement membrane [39]. Papachroni et al. have demonstrated that increased Col IV expression was linked to ovulation disorders [40]. Col III is found in the theca interna layer in the ovary [41]. Studies have shown that the expression of Col III increased markedly during luteinization [41]. Furthermore, Col III was shown to promote the proliferation of GCs [42], suggesting that its expression may support follicle development and maintenance. In line with its vital role, we found that decreased levels of Col III may contribute to ovarian functional decline. Treatment with rhCol III significantly maintained the weight of the ovaries, restored regular estrous cycles, increased the number of healthy follicles, and elevated the serum levels of E2 and AMH compared to the Age group. These findings suggested that rhCol III effectively prevented follicular atresia and delayed the decline of ovarian endocrine function.

Notably, at the 4-week time point, LH levels did not significantly differ between the rhCol III group and the Age group. LH secretion normally follows a pulsatile pattern, with the frequency and amplitude of pulses varying across different stages of the estrous cycle [43]. Although blood samples were collected at standardized time points, variations in the estrous stage among individual rats may have also contributed to the observed variability in LH measurements. Besides, factors such as stress and circadian rhythms can affect LH secretion [44], which may not have been fully controlled during the experiments. Future studies should minimize such variabilities by staging the estrous cycle and matching the timing of sample collection. In addition, 3-NTY is a stable biomarker for oxidative stress [45]. 3-NTY levels did not significantly decrease at the 4-week time point, possibly because treatment with rhCol III affected newly generated nitrative stress but did not significantly clear or reverse 3-NTY that was previously accumulated and stabilized in the ovaries.

In the follicular microenvironment, the structural integrity of the ECM is critical for follicular development. ECM not only provides structural support but also serves as a reservoir for bioactive signaling molecules that regulate follicular growth [46]. In this study, rhCol III restored aged-related alterations in the ovarian ECM. Aged ovaries exhibited a disorganized ECM structure with thickened and rigid collagen fibers. Treatment with rhCol III inhibited MMP2 expression, promoted TIMP1 expression, and stimulated the production of endogenous Col III, forming a dense and well-organized collagen network. Consistent with our findings, Ouni et al. showed that ovarian tissues from women of reproductive aged display a fine, dense collagen fiber structure compared to the ovarian tissues of postmenopausal women [47]. Rejuvenation of the ovarian ECM provided a more conducive physical and biochemical microenvironment for follicular development. rhCol III, as a humanized biomaterial, demonstrated safety in the vagina and uterus [18, 19]. Specifically considering the importance of the ovaries, we evaluated the safety of rhCol III following intra-ovarian administration in rats. Histopathological examination of major organs and assessments of liver and kidney function confirmed the safety of treatment with rhCol III.

Given the efficacy and safety of rhCol III in improving ovarian function, we investigated its direct effects on GCs. GCs play important roles in oocyte development and ovulation by facilitating signal transduction and providing nutrient transport [48]. Our study revealed that rhCol III suppressed oxidative stress, promoted proliferation, and alleviated the senescent phenotype of GCs. Energy production in GCs depends on mitochondrial oxidative phosphorylation, which can also result in ROS generation [49, 50]. The proliferation of GCs is accompanied by excessive ROS accumulation. Oxidative stress is a known driver of GC damage and follicular atresia, ultimately contributing to ovarian functional decline [51, 52]. Li et al. [53] reported that the inhibition of oxidative stress in GCs could preserve ovarian reserve.

Mechanistically, this study revealed that rhCol III primarily transmitted signals by interacting with integrin receptors on the surface of GCs. Integrins are a well-established family of transmembrane receptors that mediate cell–ECM interactions and transduce extracellular signals into specific intracellular position [54, 55]. Bioinformatic analyses revealed the involvement of the ITGA2-PI3K/Akt pathway. The experimental confirmation of reduced ITGA2 expression in GCs from older women, coupled with its positive correlation with AMH and AFC, strongly supported its role as a biomarker and potential functional mediator in human ovarian aging. A direct interaction between rhCol III and the ITGA2 receptor on GCs was confirmed via molecular docking and co-localization experiments. The PI3K/Akt pathway is a central regulator of cell proliferation, apoptosis, and metabolism [56, 57]. It modulates oxidative stress in GCs, partly through FOXO1 phosphorylation [58]. Under heat stress, bovine GCs exhibited increased levels of oxidative stress. Transcriptome analysis also indicated that the heat stress suppressed the PI3K/Akt pathway [59]. Our data revealed that rhCol III regulated GC oxidative stress in GCs via the ITGA2-PI3K/Akt signaling axis, thereby restoring ovarian function. Critically, the specific ITGA2 inhibitor E7820 completely abolished the beneficial effects of rhCol III on oxidative stress and mitochondrial function, and suppressed PI3K/Akt phosphorylation. This rescue experiment provided direct causal evidence showing that the ITGA2-PI3K/Akt signaling axis was the core mechanism underlying the beneficial effects of rhCol III.

Future studies should be conducted to investigate the broader effects of rhCol III on other ovarian cell types, including oocytes and theca cells, and further characterize downstream molecular events to support the translation of these findings into clinical practice. The long-term efficacy of rhCol III and the optimal dosage and administration frequency remain to be elucidated through further in-depth studies. Nonetheless, this work provides a foundation for ECM-based interventions in reproductive aging and female health.

## Conclusions

**Figure 9:**
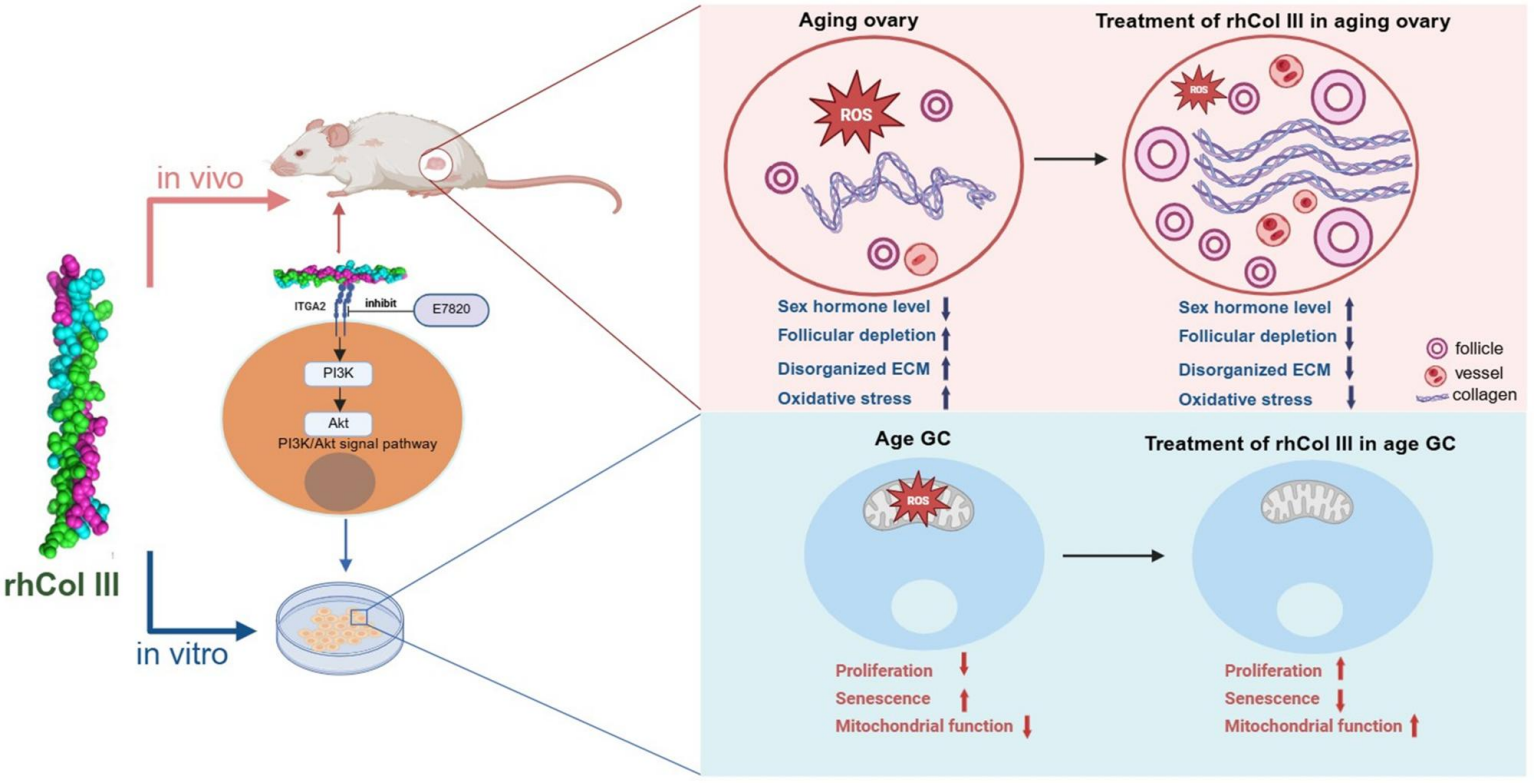
Schematic illustration showing that rhCol III restored ovarian function in aging rats and enhanced mitochondrial function in senescent GCs via the ITGA2-PI3K/akt signal pathway.

In summary, our study identified Col III deficiency as a key factor involved in ovarian function decline. We showed that treatment with rhCol III could restore ovarian function, evidenced by elevated hormone levels, reversal of follicular depletion, reconstruction of the ECM structure, and attenuation of oxidative stress in aging rats. Mechanistically, rhCol III promoted proliferation and rescued oxidative stress in GCs by activating the ITGA2-PI3K/Akt signaling pathway (Figure 9). These findings not only propose a promising biological therapeutic strategy for ovarian aging but also highlight the critical role of the ITGA2-PI3K/Akt axis, offering a novel target for future drug development.

## Supplementary Material

rbag046_Supplementary_Data

## Data Availability

The datasets used during the current study are available from the corresponding author on reasonable request.
