# Supplementary material for: Recombinant humanized type III collagen improves ovarian function via ITGA2-mediated mitochondrial function restoration in granulosa cells and extracellular matrix remodeling
Source: Regen Biomater. 2026 Mar 28;13:rbag046. doi: 10.1093/rb/rbag046 (PMC13200061; doi:10.1093/rb/rbag046)
Supplement: rbag046_Supplementary_Data [file rbag046_supplementary_data.docx]

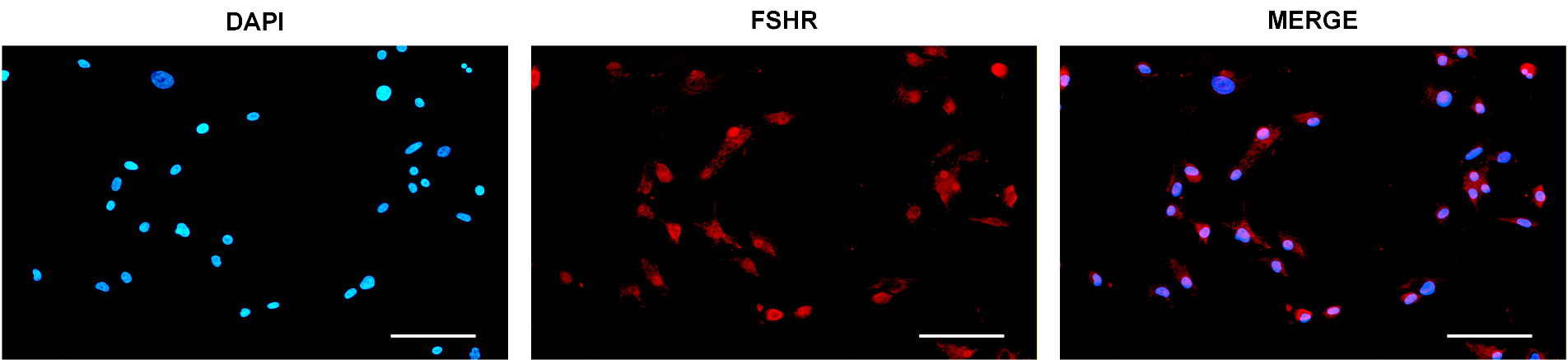


**Supplementary Figure S1** The identification of hGCs. Blue fluorescence represented nucleus. Red fluorescence represented FSHR.


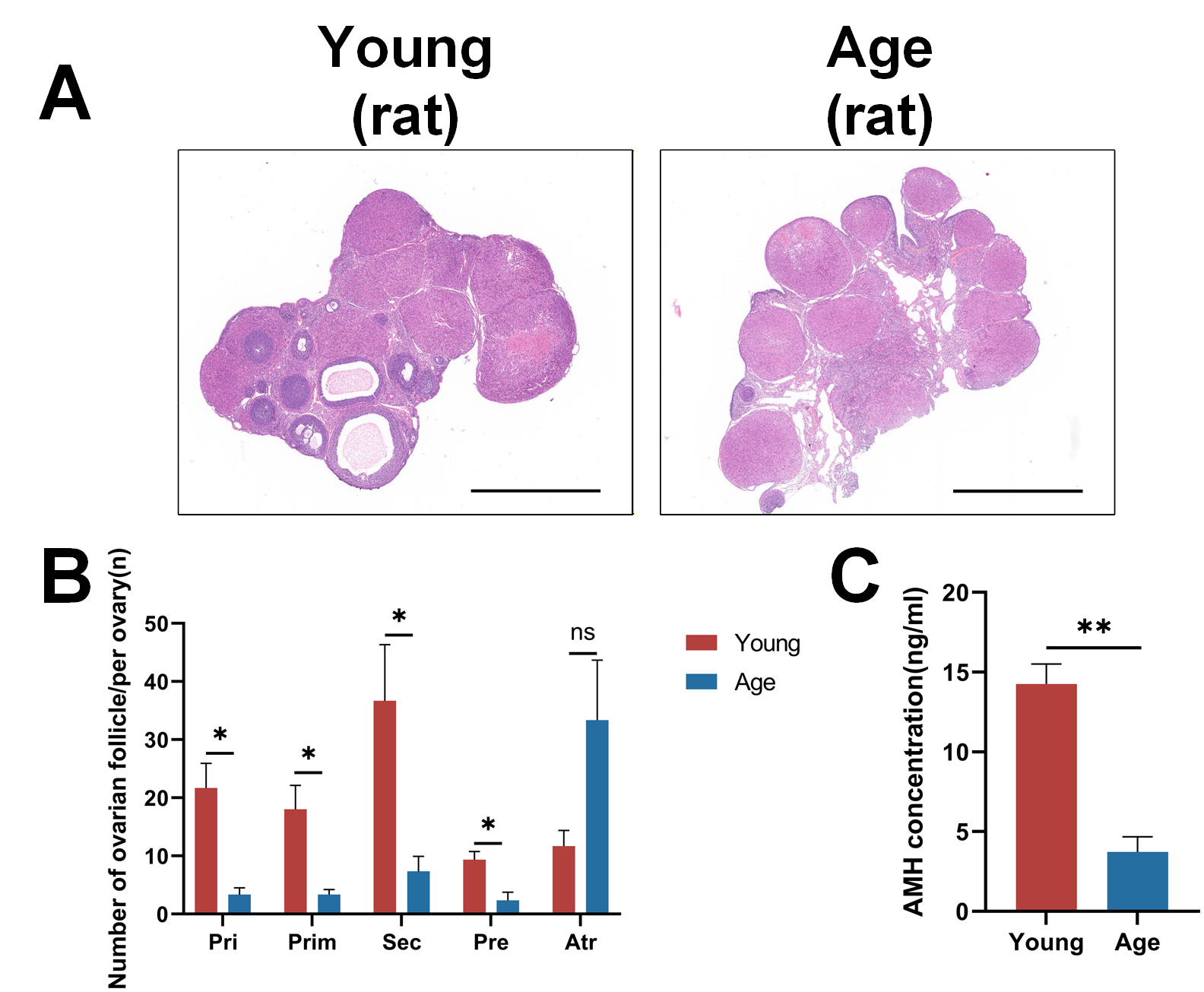


**Supplementary Figure S2** Difference between young rats and age rats. **A** Ovarian morphology was revealed by H&E staining. Scale bar=1 mm. **B** Analysis of follicle number at different stages in ovary (n=3). Pri: Primordial follicles, Prim: Primary follicles, Sec: Secondary follicles, Pre: Preovulatory follicle, Atr: Atretic follicles. **C** Serum AMH concentration of rats (n=3).


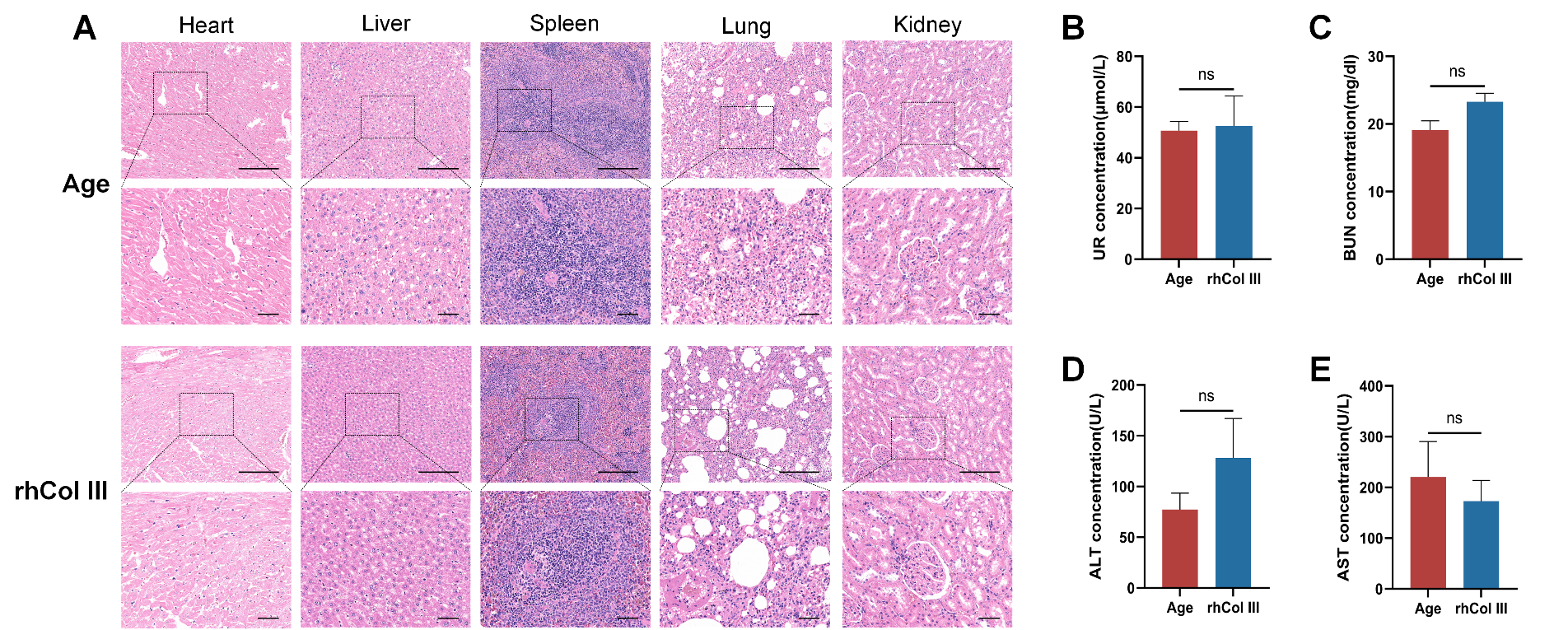


**Supplementary Figure S3** The safety of rhCOL III was assessed in animal model. **A** Typical images of tissue including heart, liver, spleen, lung and kidney between the Age group and the rhCOL III group (n=3). Scale bar = 100 µm **B** serum UR concentration of animal (n=3). **C** serum BUN concentration of animal (n=3). **D** ALT concentration of animal (n=3). **E** AST concentration of animal (n=3).


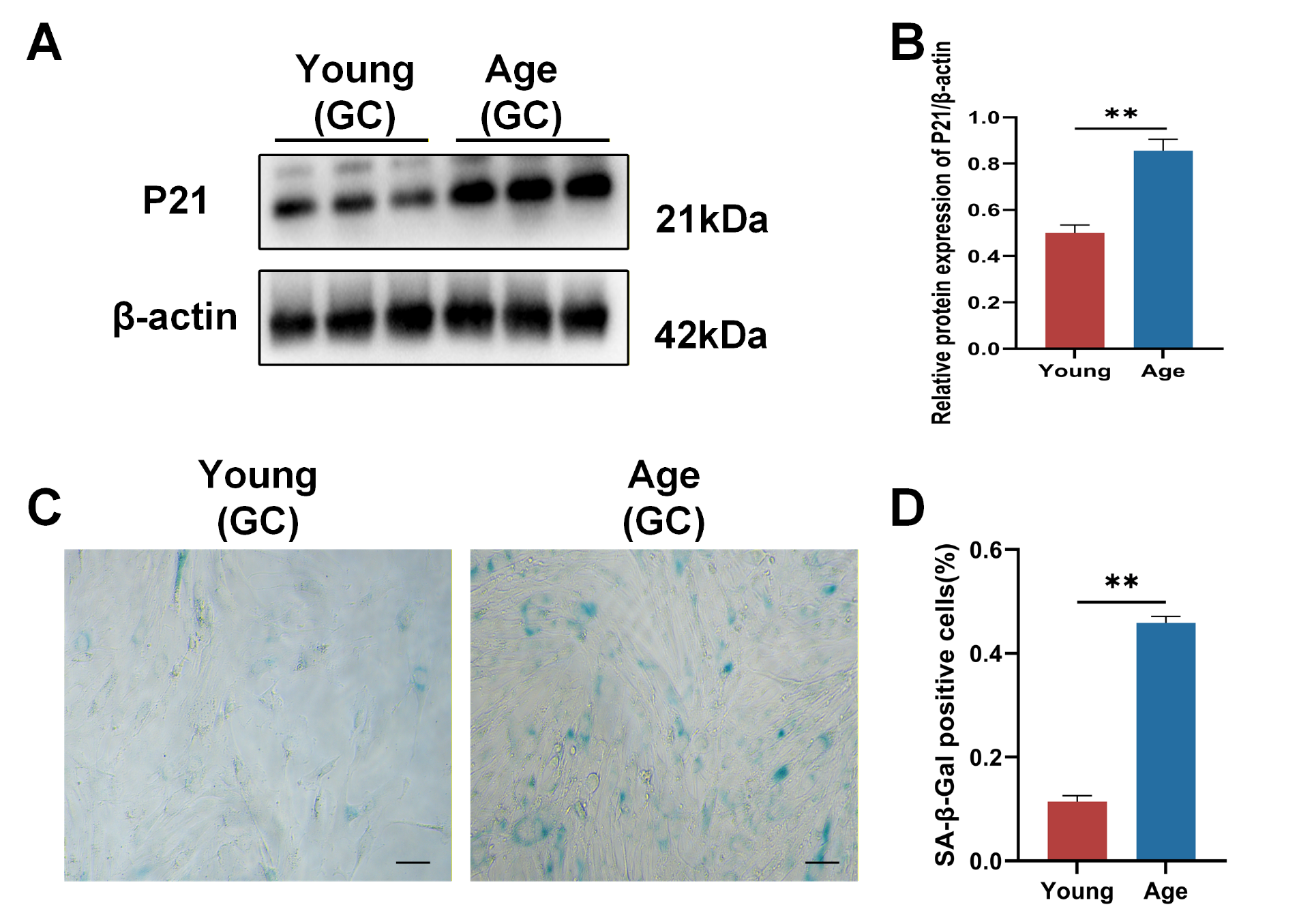


**Supplementary Figure S4** Analysis of senescent granulosa cell model. Data were representative of three independent experiments. **A** The activity of P21 were assessed by western blotting. **B** Statistical analysis of P21. **C** Cellular senescence was analysed by SA-β-gal staining. Scale bar = 50 µm. **D** statistical analysis of SA-β-gal staining.


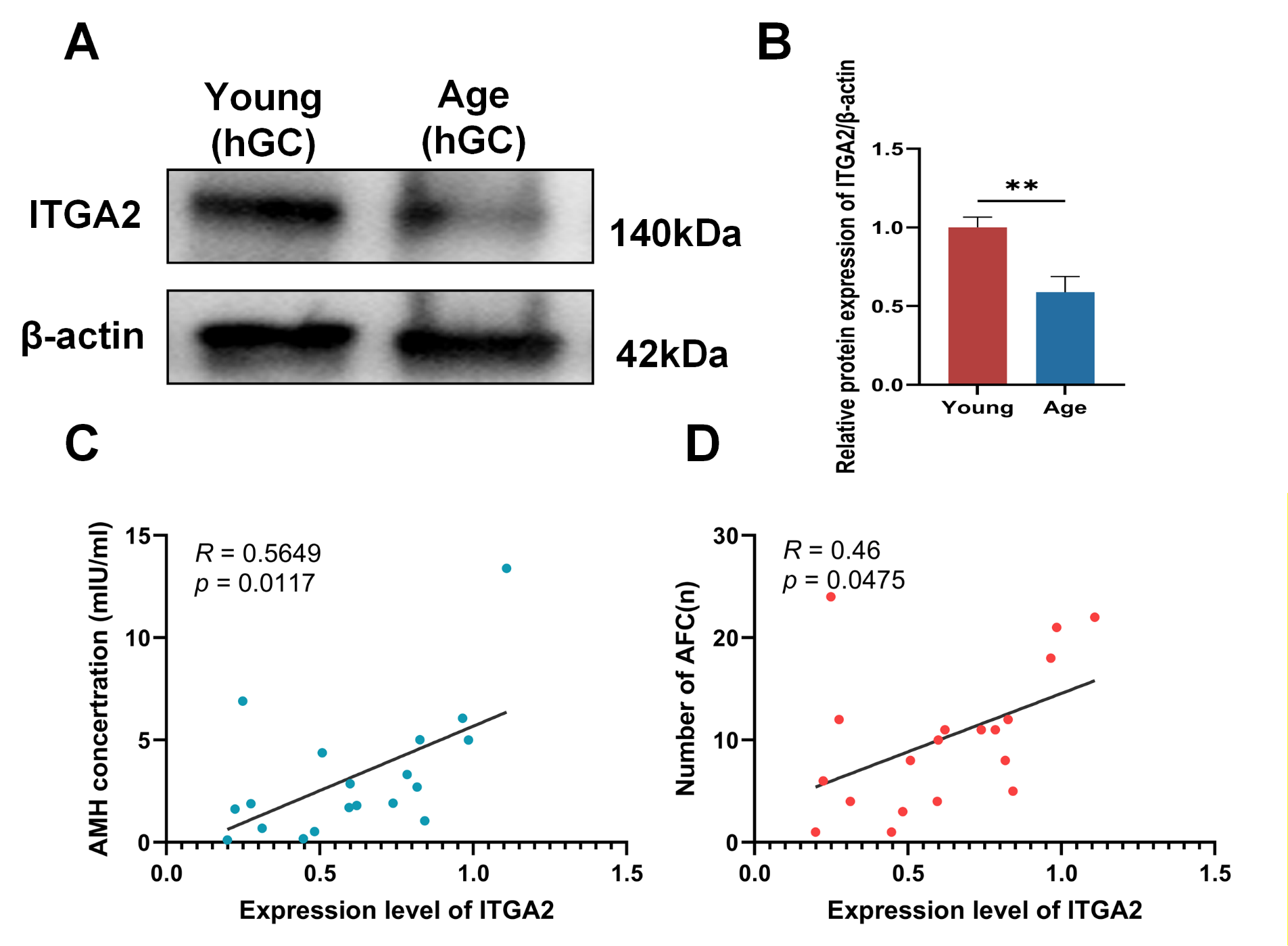


**Supplementary Figure S5** The expression of ITGA2 in human primary granulosa cell and correlation with clinical ovarian reserve. **A** The expression of ITGA2 was detected by western blotting. **B** statistical analysis of ITGA2 (n=10). **C** The correlation between the expression of ITGA2 and the level of AMH. **D** The correlation between the expression of ITGA2 and the number of Antral Follicle Count (AFC).

**Supplementary Table S1**

**Information of primary antibody**

| Target antigen | Host species | Manufacturer | Application |
| --- | --- | --- | --- |
| FSHR | Rabbit | Proteintech | IF(1:300) |
| 3-nitrotyrosine | Rabbit | MCE | IF(1:300) |
| VEGF-a | Rabbit | Proteintech | IF(1:300) |
| Collagen III | Rabbit | Proteintech | IHC(1:200)  WB(1:1000) |
| TIMP1 | Rabbit | Proteintech | IHC(1:300) |
| MMP2 | Rabbit | Proteintech | IHC(1:300) |
| P21 | Rabbit | Proteintech | WB(1:1000) |
| ITGA2 | Rabbit | Proteintech | WB(1:1000) |
| PI3K | Rabbit | Affinibody | WB(1:1000) |
| p-PI3K | Rabbit | Affinibody | WB(1:1000) |
| Akt | Rabbit | Proteintech | WB(1:2000) |
| p-Akt | Rabbit | Proteintech | WB(1:1000) |
| β-actin | Rabbit | Proteintech | WB(1:5000) |

IF: Immunofluorescence, IHC: Immunohistochemistry, WB: western blotting.
